# A computational model of the cerebellar granular layer calibrated to experimental data for studying inhibition and sensory encoding

**DOI:** 10.1038/s41598-025-25727-5

**Published:** 2025-11-25

**Authors:** María P. Tirado, Eva M. Ortigosa, Eduardo Ros, Jesús A. Garrido

**Affiliations:** Research Centre for Information and Communications Technologies (CITIC-UGR), Department of Computer Engineering, Automation and Robotics, University of Granada, Granada, Spain

**Keywords:** Computational biology and bioinformatics, Computational models, Computational neuroscience

## Abstract

The cerebellar granular layer plays a central role in sensory processing and pattern separation through its distinctive feedforward architecture. Here, we present a biologically realistic computational model of the granular layer designed to explore the functional impact of synaptic inhibition mediated by Golgi cells. The model integrates anatomical and physiological constraints to simulate realistic mossy fiber activity patterns, including spatial correlations and varying activation levels. We validate the model by replicating key findings from recent in vivo experiments, such as the role of inhibition in shaping granule cell responsiveness and the emergence of nonlinear suppression during multisensory integration. Beyond validation, the model provides a robust computational tool for studying how inhibition contributes to energy-efficient and noise-resilient sensory encoding. Mechanistic analyses revealed that moderate inhibition levels optimize pattern separation performance, with feedforward and feedback inhibitory circuits exerting distinct effects on coding expansion and decorrelation. All model code and simulation scripts are openly available, offering a framework for generating testable hypotheses and further investigating cerebellar computation and learning mechanisms in divergent feedforward networks.

**Supplementary Information:**

The online version contains supplementary material available at 10.1038/s41598-025-25727-5.

## Introduction

In the early days of neuroscience, the role of inhibition in the brain was often overlooked or disregarded, likely due to its complexity and the technological limitations of the time^1^. Researchers primarily focused on excitatory processes, which were more straightforward to study and appeared to provide direct insights into brain functions. However, in recent decades, the landscape of neuroscience has changed dramatically. Advances in electrophysiology, neuroimaging, optogenetics, and computational methods have revolutionized our ability to investigate the role of particular neural populations, enabling deeper exploration of the complex mechanisms that underlie inhibition. While classical theories^2–4^ viewed synaptic inhibition merely as a regulator of neural activity, contemporary studies—often combining experimental techniques with computational modeling—have revealed the vital role of inhibitory synapses in shaping the dynamics of brain circuits, establishing inhibition as a key contributor to learning, information processing, and cognitive control.

One domain where inhibitory circuits might play a particularly important computational role is pattern separation—a process initially explored by Marr and Albus^2,3^. They were inspired by the input layer of the cerebellar granular layer, which exhibits extensive divergence from a small number of mossy fiber inputs to a much larger population of granule cells, configuration that includes a widespread feedback inhibition that modulates granule cell excitability. Following Marr and Albus studies, multiple theories about divergent feedforward networks emerged, gaining major relevance as similar architectures as the cerebellar one can be found in many areas of the brain, such as the hippocampus^5,6^ or the olfactory bulb^7,8^. The cerebellar cortex has been therefore broadly used as an example of neuronal circuit organization that facilitates neuronal activity separation before associative learning. Importantly, computational models have highlighted several features that may contribute to pattern separation, including evidence that inhibition performs sparsening and decorrelation^9–13^. Nevertheless, the specific implications of each feature in the network remain to be fully determined.

In the cerebellar cortex, a population of inhibitory interneurons called Golgi cells modulates the activity of granule cells. Mossy fibers, which convey sensorimotor information, provide the inputs to the cerebellar granular layer, forming synapses at rosette boutons known as glomeruli with terminals of both granule and Golgi cells. Mossy fibers excite granule cells while Golgi cells receive double excitatory inputs: from mossy fibers on the basal dendrites and from parallel fibers on the apical dendrites. In turn, Golgi cells inhibit the glomeruli, creating two distinguishable loops of feedback and feedforward inhibition^14,15^. This circuitry, alongside varying population sizes, and divergence and convergence ratios, integrates signals and performs pattern separation, facilitating associative sensorimotor learning by Purkinje cells – the output neurons of the cerebellar cortex^2–4,16^. Through this connectivity architecture, the cerebellum coordinates both movements and cognitive functions effectively. However, the specific role of Golgi-cell inhibition in shaping granule cell activity—beyond its classical homeostatic function—remains underexplored, highlighting the need for computational tools to investigate its functional impact.

Although the circuitry of the cerebellar granular layer is well-established in the literature^2–4,14–16^, its dense connectivity, nonlinear synaptic interactions, and involvement in timing and learning present significant challenges for traditional experimental methods. Computational modeling that integrates realistic anatomical and physiological data is therefore indispensable for simulating and analyzing the complex neural dynamics of this layer under varying conditions, revealing principles of cerebellar processing that would otherwise be inaccessible. Some previous computational models^12,13,17–19^ offer a detailed reconstruction of the cerebellar microcircuitry, emphasizing anatomical physiological fidelity across layers. Other models^9,20–22^ prioritize abstraction and use simplified network architectures to highlight certain features—such as sparse synaptic connectivity and decorrelation—that enhance pattern separation in feedforward networks. Several other cerebellar models^11,23–25^ provide important contributions, often tailored to investigate well-defined functional hypotheses within the circuit. Validating these models against experimental data is essential to ensure their biological relevance and strengthen their predictions.

Seeking to integrate anatomically accurate reconstructions with abstract computational models, we exploited a biologically plausible computational model of the cerebellar granular layer^12,13^, together with spatially correlated input patterns in mossy fibers to achieve a more realistic scenario^26,27^. Our model was validated by simulating stimuli mimicking those used in experimental studies, testing granule cell responses under conditions with and without inhibition, and comparing our results with recent experimental data^28^. Additionally, we tested an application of our model by designing an experimental setup^20^ to examine the effects of Golgi-cell inhibition on the learning capabilities of granule cells when exposed to sets of complex, noisy patterns. Our model successfully showed that synaptic inhibition in the granular layer acts as an information filter for granule cell activity, enhancing the accuracy of pattern recognition and providing a more efficient representation of information to the subsequent layer. At a mechanistic level, we found that optimal pattern separation requires a moderate level of inhibition, with feedforward inhibition contributing to coding space expansion and feedback inhibition promoting decorrelation of activity patterns. In this work, we therefore introduce a validated computational tool, openly accessible to the scientific community, for further exploration of the mechanism underlying cerebellar processing and learning.

## Results

### Modeling the cerebellar granular layer

**Fig. 1:**
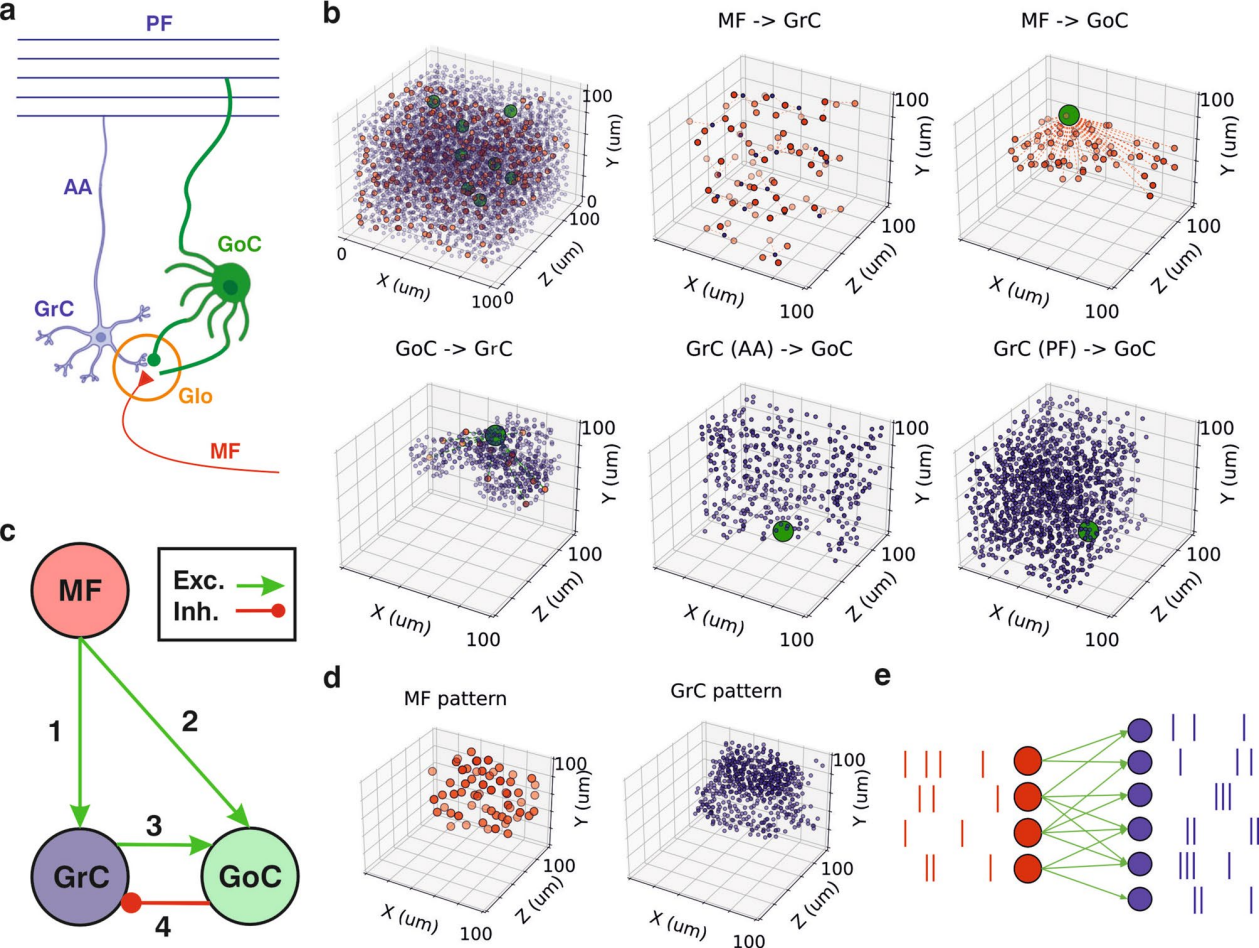
Modeling the cerebellar granular layer. (**a**) Schematic of biological connections taking place in the glomerulus. (**b**) Top left, built anatomical model in a cube of side 100 µm. Top center, excitatory convergence of mossy fibers (red dots) to granule cells (blue dots). Top right, excitatory convergence of mossy fibers to a single Golgi cell (green dot). Bottom left, inhibitory convergence of a single Golgi cell to glomeruli and their connected granule cells. Bottom center and right, excitatory convergence of granule cells to Golgi cells via the ascendant axons and the parallel fibers, respectively. (**c**) Simplified diagram of the connections between the neuron populations. (**d**) Left, active mossy fibers and right, granule cells in a pattern example (σ = 40 µm). (**e**) Schematic of the spiking network model for the MF—GrC synapsis with sample spike trains.

The cerebellar granular layer is the recipient of inputs from mossy fibers (MFs), which terminate in large rosette-like structures known as synaptic glomeruli (Glos). These glomeruli consist mainly of the mossy fiber terminals, dendrites of granule cells (GrCs) and both dendrites and axons of Golgi cells (GoCs). Golgi cells regulate the glomeruli with inhibitory signals, while information is passed on to the granule and Golgi cell from the mossy fiber (Fig. 1a,c). To capture these synaptic connectivities, we built a 3-dimensional model of a local region of the cerebellar granular layer, according to realistic neuroanatomical models^12,13^ with point neurons. Within a cube of side 100 µm and following anatomical constraints, the number of cells generated and positioned were around 300 MFs, 3700 GrCs and 8 GoCs. Connectivity-wise, four types of synapses were taken into consideration: the inhibitory synapse GoC—GrC and the excitatory synapses MF—GrC, MF—GoC and GrC—GoC. This last synapse was divided into two different paths: via the ascending axons (AA) and the parallel fibers (PF) (Fig. 1b). The circuit from MF to GoC, providing excitation, followed by inhibition from GoC to GrC, constituted the feedforward inhibitory loop. In contrast, the feedback loop involved excitation from MF to GrC, then from GrC to GoC, leading to delayed inhibition back onto GrC. We assessed the biological plausibility of the model by comparing convergence and divergence ratios with known experimental values and taking into consideration densities and radii of the different neural populations (see Supplementary Materials and Supplementary Fig. S1).

To define the network activity input via mossy fibers, we used a technique that generates binary spike trains characterized by predetermined firing rates and spike correlations^26^. This approach enabled us to capture spatial correlations in MF activity patterns instead of relying on randomly uniform activity distributions (Fig. 1d). A Gaussian correlation function was used to describe the distance-dependence of rosette co-activation, which was parameterized by its standard deviation σ (the correlation radius). Simulations were carried out using the EDLUT simulator^27^, which allows for fast time-driven simulations of medium-size spiking neural networks (Fig. 1e). During simulations, the default firing rate for active MFs was set to 50 Hz using a Poisson generator while inactive MFs remained silent. GrCs and GoCs were modeled using the Leaky Integrate-and-Fire (LIF) model through a differential equation that accounts for the effect of chemical synapses (including α-amino-3-hydroxy-5-methyl-4-isoxazolepropionic acid (AMPA) and gamma-aminobutyric acid (GABA) receptors) and resting conductances (see Methods). In order to make the model as simple as possible, synaptic weights were constant parameters for every simulation, meaning there was no synaptic plasticity. The synaptic weights took an important part in the model validation, as different values resulted in modified modulation of the output activity, enabling us to study the impact of different synaptic weights (see Methods).

### Model validation against experimental findings

To ensure the biological plausibility and relevance of the model, we first validated it by replicating several key experimental observations reported in recent in vivo studies, particularly those by Fleming et al. (2024). These experiments investigated the role of Golgi cell-mediated inhibition in shaping granule cell activity in response to auditory and somatosensory stimuli.

Using spatially correlated mossy fiber input patterns with parameters matched to the experimental protocols, we simulated stimulus-evoked activity in granule cells under both control and disinhibited conditions (mimicking DART infusion). We analyzed population response probability, single-trial amplitude, and stimulus selectivity to determine whether the model could capture the essential features of granule cell encoding under varying levels of inhibition.

Additionally, we examined how the model reproduces nonlinear effects observed during multisensory integration, such as the emergence of responses to combined stimuli that are not present for the individual components, and the suppression of expected responses under inhibitory control. These simulations demonstrate that the model can reproduce both quantitative and qualitative aspects of experimental data, establishing it as a reliable platform for further computational exploration.

#### Synaptic inhibition reshapes MF-GrC connectivity for an enhanced multisensory pattern representation in GrCs

**Fig. 2:**
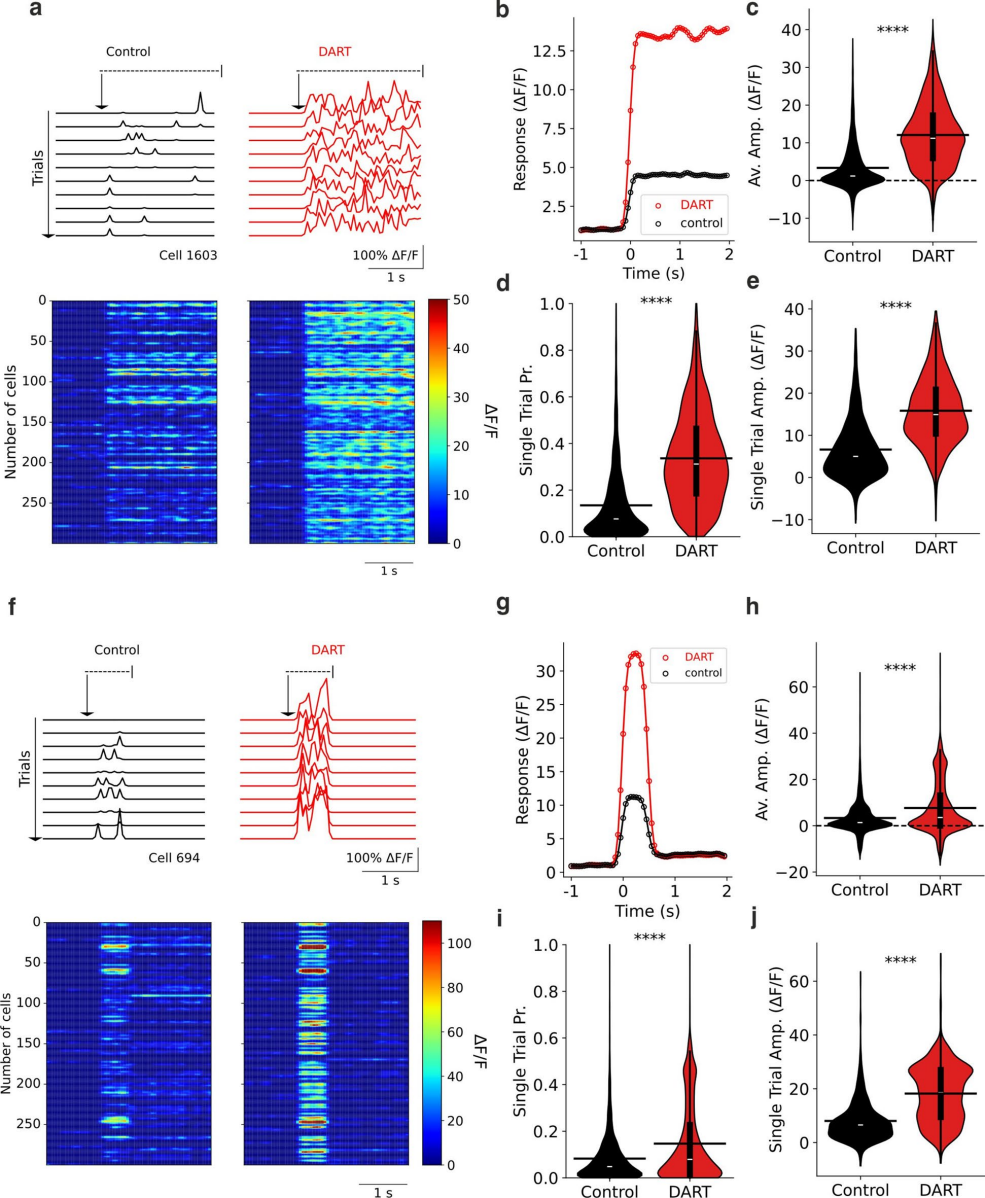
Local synaptic inhibition sparsens and thresholds cerebellar granule activity. (**a**) Top, example of firing rate activity from a granule cell on simulated auditory pattern presentation trials for the control state (black) and the DART state (red) where inhibition is blocked. Bottom, mean activity of some cells with significant responses to a pattern presentation for the control state (left) and the DART state (right). (**b**) Mean time course of responses during a pattern presentation for the control state (black) and the DART state (red) (n = 3760). (**c**) Mean response amplitudes for individual cells in control (black) and DART (red) state (n = 3760). (**d**) Mean firing probabilities for individual cells during a single trial in control (black) and DART (red) state (n = 3760). (**e**) Mean response amplitudes on all trials for cells with significant responses in control (black) and DART (red) state (n = 1690). (**f**–**j**), same as a-e for responses to simulated somatosensory stimuli (**f**–**i**, n = 3760; **j**, n = 1271). ****P < 0.0001, paired *t* test.

Therefore, our first goal was to measure how discrete sensory inputs are encoded in the granule cell layer and how they could be used as an initial substrate for associative learning, reproducing recent experimental results in the literature^28^. To replicate sensory-evoked auditory stimulation (tones in experimental results^28^) we selected random patterns with a medium rate of active MFs (45%) with individual firing rate of 50 Hz and low spatial correlation (σ = 5 µm) (Fig. 2a,b) (see Methods section for additional details on stimulation). These patterns were presented during 3 s. In contrast, to replicate somatosensory stimuli (air-puffs in experimental results^28^), we generated shorter (500 ms) patterns with slightly lower rate of active MFs (35%), increasing the firing rate of active mossy fibers to 100 Hz (Fig. 2f,g). These patterns were also more spatially correlated (σ = 40 µm) in order to target more specific granule cell ensembles. Before pattern presentation, noise was presented during the first second of simulation, defining noise as a low fraction (10%) of randomly chosen active MFs. As an equivalent to ΔF/F (change in fluorescence divided by baseline fluorescence), we studied the difference of firing rate considering sliding windows of 50 ms divided by the base average firing rate before pattern presentation. Each simulation was conducted under two conditions: first one enabling inhibition, representing the control state, and second one disabling inhibition to mimic DART (drug acutely restricted by tethering) infusion, i.e. the blocking of synaptic inhibition onto GrCs, which was executed by setting inhibitory weights of GoC—GrC to zero. This stimulation protocol was repeated across 10 trials changing the Poisson generator seed to favor randomness. Results obtained were very similar for different patterns that followed the before-given characteristics.

For both (auditory and somatosensory) stimulation types, granule cells in the presence of inhibition produced responses in granule cells ensembles with lower probabilities (Fig. 2d,i; auditory control: 0.13 ± 0.00; somatosensory control: 0.08 ± 0.00, *P* < 0.0001, paired *t* test) and smaller single-trial amplitudes (Fig. 2e,j; auditory control: 6.62 ± 0.17 ΔF/F; somatosensory control: 8.03 ± 0.22 ΔF/F, *P* < 0.0001, paired *t* test). The DART infusion replication produced significantly larger mean population responses (Fig. 2b,c,g,h), with increased response probabilities (Fig. 2d,i; auditory DART: 0.34 ± 0.00; somatosensory DART: 0.15 ± 0.00, *P* < 0.0001, paired *t* test) and increased amplitudes of single trial responses (Fig. 2e,j; auditory DART: 15.83 ± 0.19 ΔF/F; somatosensory DART: 18.21 ± 0.30 ΔF/F, *P* < 0.0001, paired *t* test). As previously reported in experimental results^28^, many granule cells that were silent in control conditions became responsive after blocking synaptic inhibition, resulting in a dramatic increase in the number of granule cells responsive to both auditory and somatosensory stimulation. On average, there was a large expansion in fraction from control to DART conditions for auditory stimuli (14.5 ± 0.1% GrCs, control; 45.3 ± 0.2% GrCs DART), while for somatosensory stimuli the expansion was smaller (17.7 ± 0.5% GrCs, control; 31.8 ± 0.2% GrCs DART). These percentages of active granule cells in control and DART conditions were given by the chosen input fraction (45% of active MFs for auditory stimuli and 35% for somatosensory stimuli) and the calibrated weight between mossy fibers and granule cells in the model (see Methods).

**Fig. 3:**
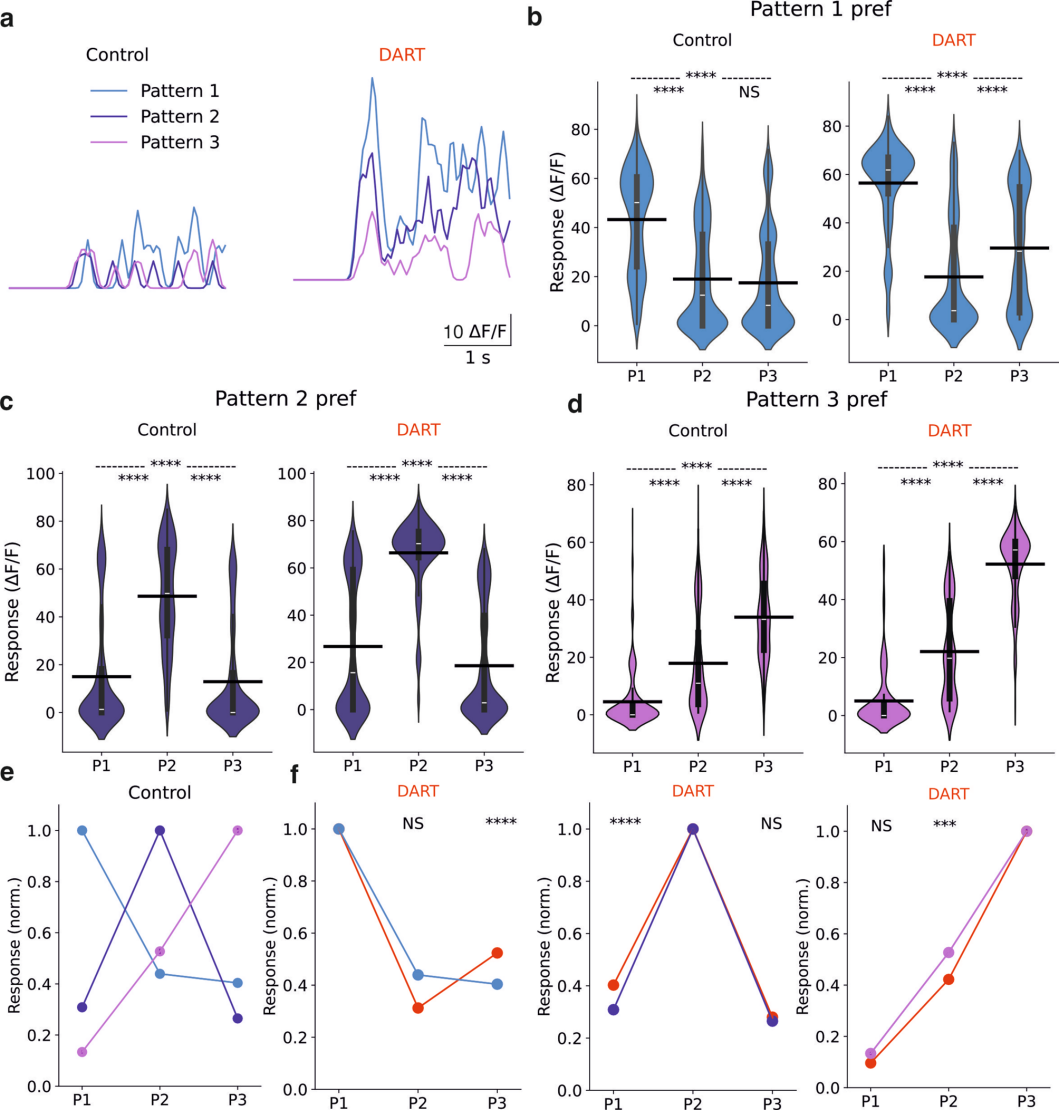
Cerebellar granule cells exhibit stimulus feature preferences that are not abolished by blocking synaptic inhibition. (**a**) Average firing rate for an example cell in response to three different stimuli of similar input activity fraction. The cell responds preferentially to the first pattern before (left) and after (right) DART. (**b**) Maximum responses for cells significantly responsive that prefer pattern 1 (n = 1183 control, n = 792 DART). (**c**) Same as (**b**), for cells preferring pattern 2 (n = 1298 control, n = 1842 DART). (**d**) Same as b, for cells preferring pattern 3 (n = 525 control, n = 415 DART). (**e**) Normalized responses in control conditions for all granule cells grouped according to the pattern that drove the maximum response. (**f**). Same as e, for granule cells before and after DART infusion. *P < 0.05, **P < 0.01, ***P < 0.001, ****P < 0.0001, paired *t* test. *NS*, not significant.

Once verified that each discrete stimulus evokes sparse granule cell activity under the presence of inhibition, we then aimed to determine whether individual granule cells in our model exhibited selective responses to specific stimuli, as outlined in experimental results^28^. In this experiment, we selected three distinct patterns with a similar proportion of active MFs (65%) and high spatial correlation (σ = 40 µm). The spatial correlation of input patterns for this experiment had little influence on the outcome, as similar results were obtained across different correlations levels. Random activity was conveyed to the MFs during the first second of simulation and, right after that, one of the three patterns was presented during 2 s. This protocol was repeated for each pattern and the resulting activity in the GrCs was analyzed (Fig. 3a). We found that GrCs displayed preferences for specific stimulus features, since those cells that preferred (more intense responses to) a given pattern produced diminished responses for the remaining two (Fig. 3b–d). While blocking synaptic inhibition accentuated stimulus preferences, mean population preferences still remained significantly high for those cases (Fig. 3e,f). These data support that granule cell stimulus preferences are based on presynaptic mossy fiber input structure, consistent with the small number of MFs converging into every granule cell and the lack of recurrent excitatory processing among GrCs, in agreement with^13^. According to these results, recurrent inhibition would not directly affect the preference of certain neurons for specific input patterns.

However, it is known that some GrCs receive MFs input from more than one sensorial source and it is thought that the integration of these inputs could enhance the diversity of GrCs encoding. To explore the nature of this integration and the presence of multisensory ensembles in our model, we examine population responses to overlapping activity patterns, following counterpart experimental set-ups^28^. To ensure overlapping between active neurons, we use patterns of low spatial correlation (σ = 5 µm). Additionally, we combined patterns of 35% and 45% MF active fractions, in order to again mimic auditory and sensorimotor stimuli, as seen in the previous sections. The stimulation protocol of the simulation was designed to sequentially present the first pattern, then the second pattern and finally the combination of both patterns. This procedure was repeated across 10 trials, with consistent results for patterns with similar parameters as the previously described. GrC responses to the simultaneous presentation of both stimuli were categorized into four types: *facilitated* if the mean response is higher than to individual stimuli, *unchanged* if similar, *suppressed* if lower, and *emergent* if substantially higher (See Methods).

**Fig. 4:**
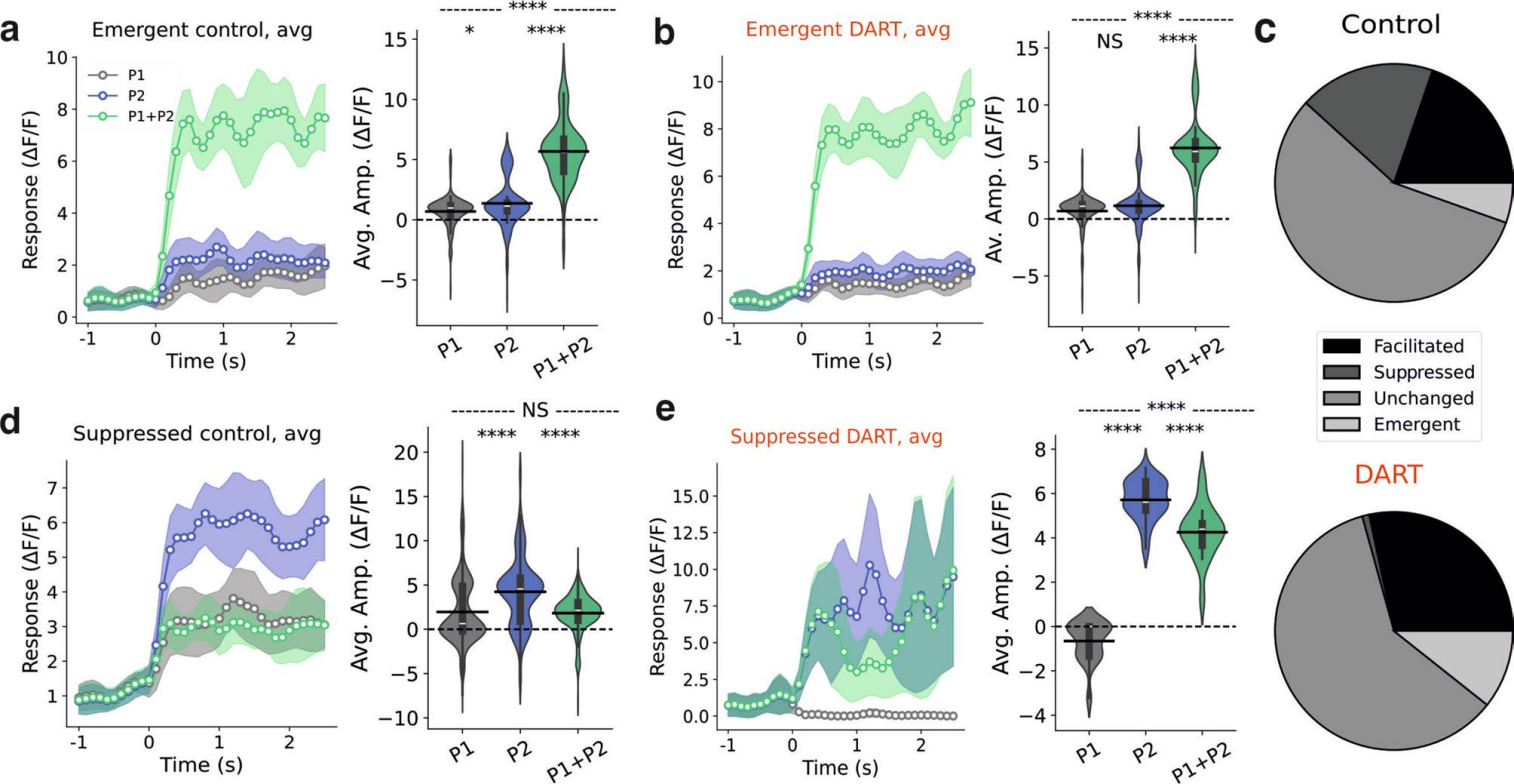
Coincident stimuli create unique granule cell ensembles in presence of GoC inhibition. (**a**) Left, time course of responses to unisensory (pattern 1, gray and pattern 2, blue) and multisensory (patterns 1 + 2, green) stimuli for all emergent cells. Right, amplitude of response to the three situations for all matched emergent cells in the control state. (**b**) Same as a, for matched emergent cells in the DART state. (**c**) Pie charts illustrating the relative prevalence of each response category before (top: facilitated, n = 792; unchanged, n = 2124; suppressed, n = 656 and emergent, n = 188) and after (bottom: facilitated, n = 1111; unchanged, n = 2265; suppressed, n = 8 and emergent, n = 376) DART infusion. (**d**) Same as a, for matched suppressed cells in the control state. (**e**) Same as a, for matched suppressed cells in the DART state. *P < 0.05, **P < 0.01, ***P < 0.001, ****P < 0.0001, one-way ANOVA. *NS*, not significant.

Combining patterns of different MF active fractions led us to reproduce the two main findings from experimental results^28^. We observed that 5% of GrCs in control cases and 10% of GrCs in DART cases that did not respond significantly to either stimuli individually, became active when exposed to the combined stimuli. These GrCs were identified as emergent cells (Fig. 4a). These results suggest that the integration of the MF activity for both stimuli in the preceding layer can drive new GrCs above the spike threshold, thereby forming new multisensory ensembles that were not apparent in isolated stimuli. Notably, this phenomenon was evident even under the absence of inhibition in DART cases (Fig. 4b). Another replicated observation in our model is the presence of suppressed cells (Fig. 4d). As expected, the number of suppressed cells was significantly higher in control cases (17.4% of cells) compared to cases involving DART infusion (0.2% of cells), where in the majority of trials we found this group to be absent (Fig. 4e). This effect therefore further distinguishes the new multisensory ensemble from the unisensory ensembles. Differently from the experiments reported in^28^ we found that the majority of the cells remained unchanged (56.4% in control conditions and 60.2% in DART condition) (Fig. 4c). However, this difference could emerge from the different methodology to classify neurons as emerging or suppressed.

### Functional assessment: pattern separation, inhibition and encoding performance

Having validated the model against experimental data (Figs. 2–4), we next sought to assess its ability to reproduce core computational principles associated with cerebellar granular layer function. Specifically, we evaluated whether our model supports pattern separation and whether inhibitory modulation and varying input conditions affect classification performance and noise robustness.

These simulations do not aim to present novel biological findings, but rather serve to demonstrate that the model captures key theoretical expectations from classical and recent cerebellar literature. Granule cells are hypothesized to transform mossy fiber input into sparse, decorrelated representations that facilitate associative learning in Purkinje cells. Moreover, Golgi cell-mediated inhibition is proposed to enhance this process by suppressing redundant activity and increasing coding efficiency.

Using a simple linear classifier, we tested whether the output activity of the model under different levels of mossy fiber input and inhibition could support reliable pattern discrimination. Additionally, we introduced structured noise to evaluate the model’s behavior under degraded input conditions. These results provide further support for the model’s functional validity and its utility in computational studies of cerebellar encoding and learning.

#### Complex and highly active MF stimulation facilitates pattern recognition in GrC ensembles

**Fig. 5:**
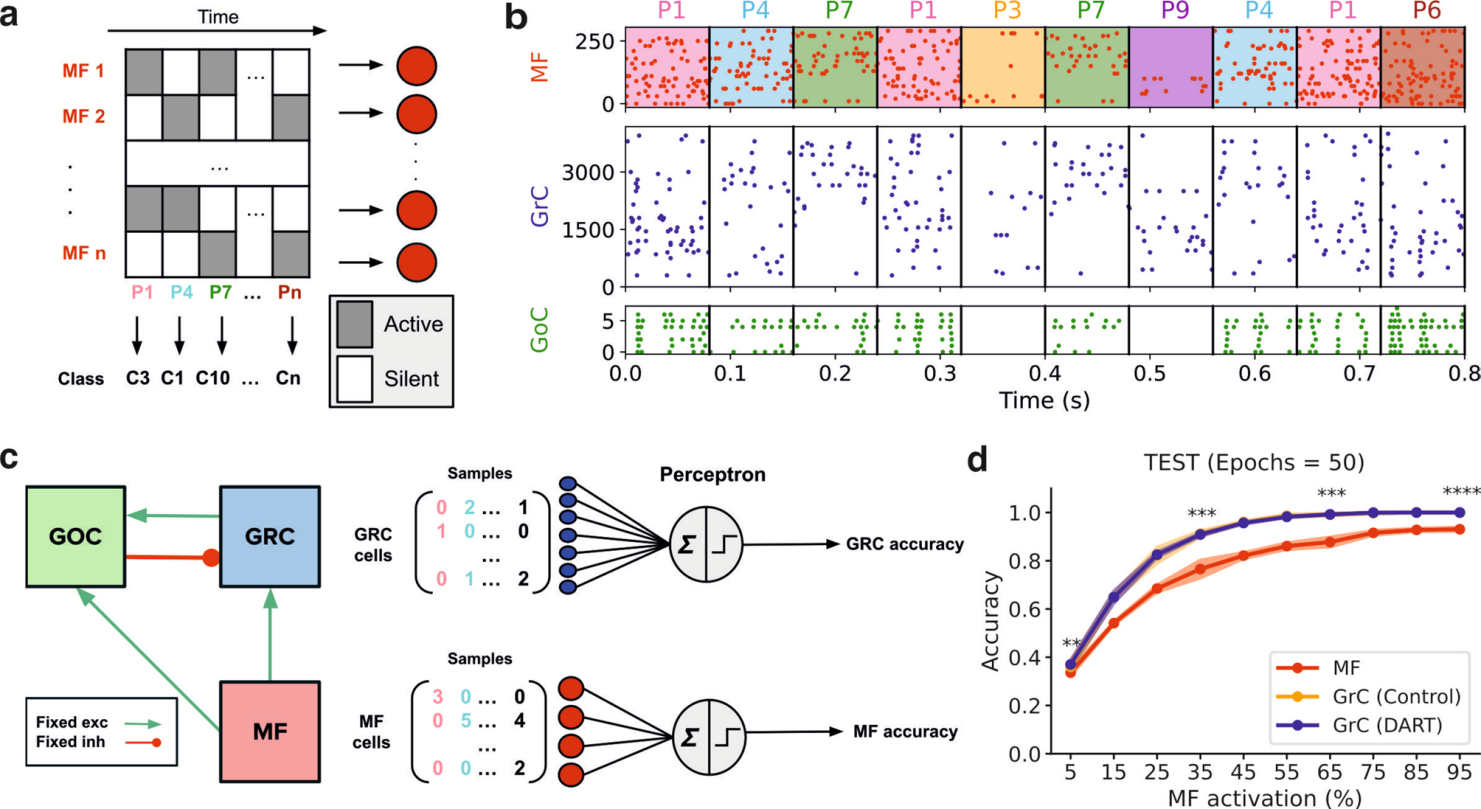
Model set-up to study pattern recognition in the granular layer. (**a**) Schematic of the binary input patterns in the population of mossy fibers. Each pattern is randomly assigned to a class among 10 different classes. (**b**) Sample of spike trains during 800 ms of simulation showing the activity of the neurons for 10 different pattern presentations. For simplification of the plot, we only show the activity of 30 MFs and 75 GrCs randomly selected. (**c**) From left to right, schematic diagram of the granular layer network, example of spikes count collected in matrices separately for GrC and MF and schematic of the single layer perceptron decoder of the activity. (**d**) Example of the average accuracy results for the test set at the end of 50 training epochs as a function of the average MF activation percentage of the set of patterns. Shading indicates the 95% confidence interval (CI) for each point. **P < 0.01, ***P < 0.001, ****P < 0.0001, paired *t* test results for GrC (DART).

Once our model validation confirmed the experimentally reported behavior of granule cells under local synaptic inhibition from GoCs, we moved on to explore whether the granular layer topology facilitates pattern representation, particularly focusing on the role of inhibition in shaping these processes. Aiming to estimate the efficiency of associative learning occurring in the Purkinje layer, we trained a perceptron to recognize the GrC response to stimulation patterns^20^. Thus, we generated a series of 640 binary spatially correlated patterns defined by a correlation radius of σ = 40 µm and varying average MF active fractions spanning from 0.05 to 0.95, which served as our input patterns (Fig. 5a). Each randomly selected pattern was presented for 80 ms, with active MF firing at 50 Hz, resulting in diverse firing patterns in both GrCs and GoCs (Fig. 5b).

After the simulations, spike counts were collected for each neuron population (MF and GrC) across all patterns, resulting in the compilation of two spike count matrices. These matrices served as input for our classifier, a single-layer perceptron (Fig. 5c). The perceptron classifier aimed to simulate Purkinje cells associative learning in the subsequent Purkinje layer. Given the limited understanding of which features of GrC patterns are critical for Purkinje cell learning, we opted for random classification to assess general pattern separation. Therefore, each input pattern was randomly assigned to one of 10 different classes (Fig. 5a). Patterns were presented multiple times during simulations and the collected dataset of samples were divided into training (70%), validation (10%), and test (20%) sets (Fig. 5c).

Our tests consistently showed that patterns were more accurately recognised when using granular layer activity than when using MF activity (*P < 0.05 for MF activation 5%). This improvement was particularly evident when the patterns were stimulated with higher and more complex activity, characterized by increased input activity fraction and highly spatially correlated patterns (***P < 0.001 and ****P < 0.0001 for MF activation 35%, 65% and 95%) (Fig. 5d). According to these results, even with only 50 training epochs, a linear classifier is able to recognize and classify 640 GrC patterns with 0.9 accuracy if the MF activation goes above 25%. These results support experimental evidence that physical activity, understood as simultaneous stimuli presentations of high input activity, enhances associative learning in one cerebellum-dependent behavior^29,30^. In our experiment, we also considered the DART scenario, where inhibition was completely blocked (Fig. 5d) and two additional cases with different levels of inhibition achieved through increasing excitatory weights of MF—GoC and GrC—GoC synapses (Supplementary Fig. S3a). However, the results for both the DART and increased inhibition cases were very similar, prompting us to explore whether increasing task difficulty might reveal any performance difference at all.

#### Synaptic inhibition facilitates pattern separation and energetically efficient representation for GrC ensembles

**Fig. 6:**
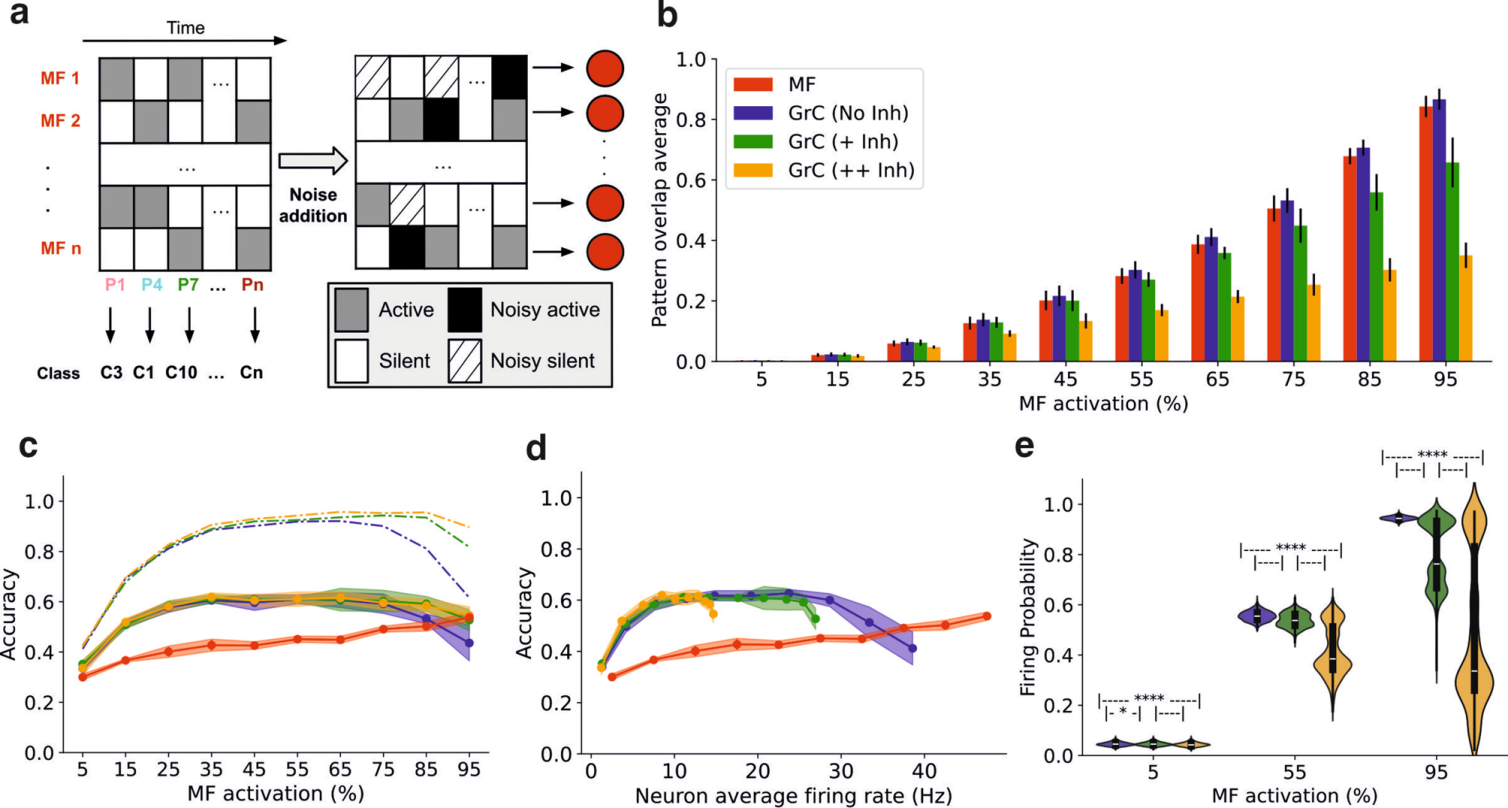
Inhibition facilitates energetically efficient representation for GrC ensembles in the presence of noisy stimuli (20% of noisy cells). (**a**) Schematic of the noise implementation in the binary input patterns driving the population of mossy fibers. (**b**) Average overlap between active cells of different patterns for mossy fibers (red), granule cells with no inhibition (DART) (blue), with a low level of inhibition (green) and with a high level of inhibition (yellow) (See Methods for further information on inhibition levels). (**c**) Accuracy for the test set after 500-epoch-learning as a function of the input active fraction for mossy fibers and different levels of inhibition. Shading represents the confidence interval (95%). Dash-dotted lines represent the accuracy obtained with the training set with different levels of inhibition. (**d**) Accuracy test results after 500-epoch-learning as a function of the GrC and MF average firing rate and different levels of inhibition. Shading represents the confidence interval (95%). (**e**) Average GrC firing probability distribution across all the patterns for each configuration for 5, 55 and 95% of active MF. *P < 0.05, ****P < 0.0001, paired *t* test.

Therefore, we aimed to increase the complexity of the classification task by introducing noisy patterns and observing whether different inhibition settings preserved accurate classification. The implementation of noise in the model consisted of selecting a percentage of random MF for each pattern presentation, and re-setting their activity, with a probability of being active given by the average MF active fraction of the patterns set (See Methods). Following this method, some active and silent neurons from the pattern would preserve their state while others would become *noisy* silent or *noisy* active (Fig. 6a). The average fraction of activity of the set of patterns was preserved, despite the introduction of noise. In our experiment, we consider a percentage of 20% noisy MF, indicating that the 20% of the cells randomly changed their firing state for every presentation of the pattern. Consequently to noise introduction, the larger the noise percentage was chosen, the less accurate was the perceptron classification performance, as shown in Supplementary Fig. S3c, d.

Our tests revealed that learning from GrC representations exhibits more accurate classification in comparison to learning from noisy MF representations of patterns (Fig. 6c). *P*-values for paired *t* tests between GrC configurations are non significant (NS) except for very high MF activation, where *P* value is less than 0.5 (see Supplementary Fig. S3b). Although for low and medium levels of MF activation there is no significant difference between learning from *inhibited* or DART representations, higher levels of MF activation reveals higher accuracy on inhibited representations (Fig. 6c; For 95% active MF, GrC (No Inh): 0.44, *CI*: [0.36, 0.50]; GrC (+ Inh): 0.53, *CI*: [0.49, 0.57]; GrC (+ + Inh): 0.55, *CI*: [0.51, 0.58]; *P* value < 0.5, paired *t* test). This phenomenon may emerge from wider GoC axons than dendrites, resulting in enhanced inhibition over non-locally-correlated noisy GrCs, filtering the original pattern and reducing overlap between patterns (Fig. 6b) (See Supplementary Fig. S5 for visualizations of GoC inhibitory effect).

Based on these results we wondered if the accuracy of learning depended on the average activity being learned by the classifier. Notably, considering the average firing rate of the neuron population during simulations, we observed that inhibition leads to a more energetically efficient representation of information. According to our results, higher GoC thresholding (+ Inh and +  + Inh) reduced GrC firing rates while maintaining similar levels of classification accuracy than higher firing rates in DART condition (Fig. 6d). These simulations provide evidence that GoC inhibition effectively reduces GrC activity while avoiding loss of information and reducing pattern overlapping. We found that the firing probability distributions for GrCs during simulations get more sparse in the presence of inhibition as the MF activation increases (Fig. 6e), which again shows the key role of GoCs in context with very dense inputs and results in more distinguishable patterns for the classifier (Fig. 6c).

To further investigate the role of inhibition in cerebellar processing, we aimed to study if GoC inhibition could impact the timing of GrC activity in our model, emulating experimental set up defined in^28^. The experiment consisted in a stimulus presentation during 500 ms after a previous phase of 250 ms with a low fraction (10%) of random active noise, presenting the stimulus in two rounds of 10 trials each. The stimulus was a highly MF active pattern (95%) with 20% of noise, with an inhibition level set to threshold “+  + Inh ” (see Methods). We found that peaks of mean responses were not temporally consistent across trials (Supplementary Fig. S6a). Computing the GrC spiking probability in two rounds of trials, we observed a front of first spikes in the first round that faded in the second round of trials for both control and DART cases (Supplementary Fig. S6b, c). These findings align with experimental results^28^ and indicate that synaptic inhibition alone may not regulate the temporal consistency of granule cell activity, implying the involvement of additional neural mechanisms.

Additionally, we aimed to reproduce previous findings^28^ showing that inhibition is necessary not just for learning but also for expressing learned behaviour. To test whether our model exhibits a similar dependency, we trained the classifier with intact inhibition and we then evaluated the performance on a subset of patterns with and without inhibition. Blocking inhibition after learning led to a clear drop in accuracy (Supplementary Fig. S7), mirroring experimental findings. This effect was present even at low levels of input activation and increased more with stronger input. However, while our task featured stimulus complexity, it shall be noted that it lacked the temporal dynamics of eyeblink conditioning, where behavioural responses are delayed.

### Mechanistic analysis of inhibition in pattern separation

**Fig. 7:**
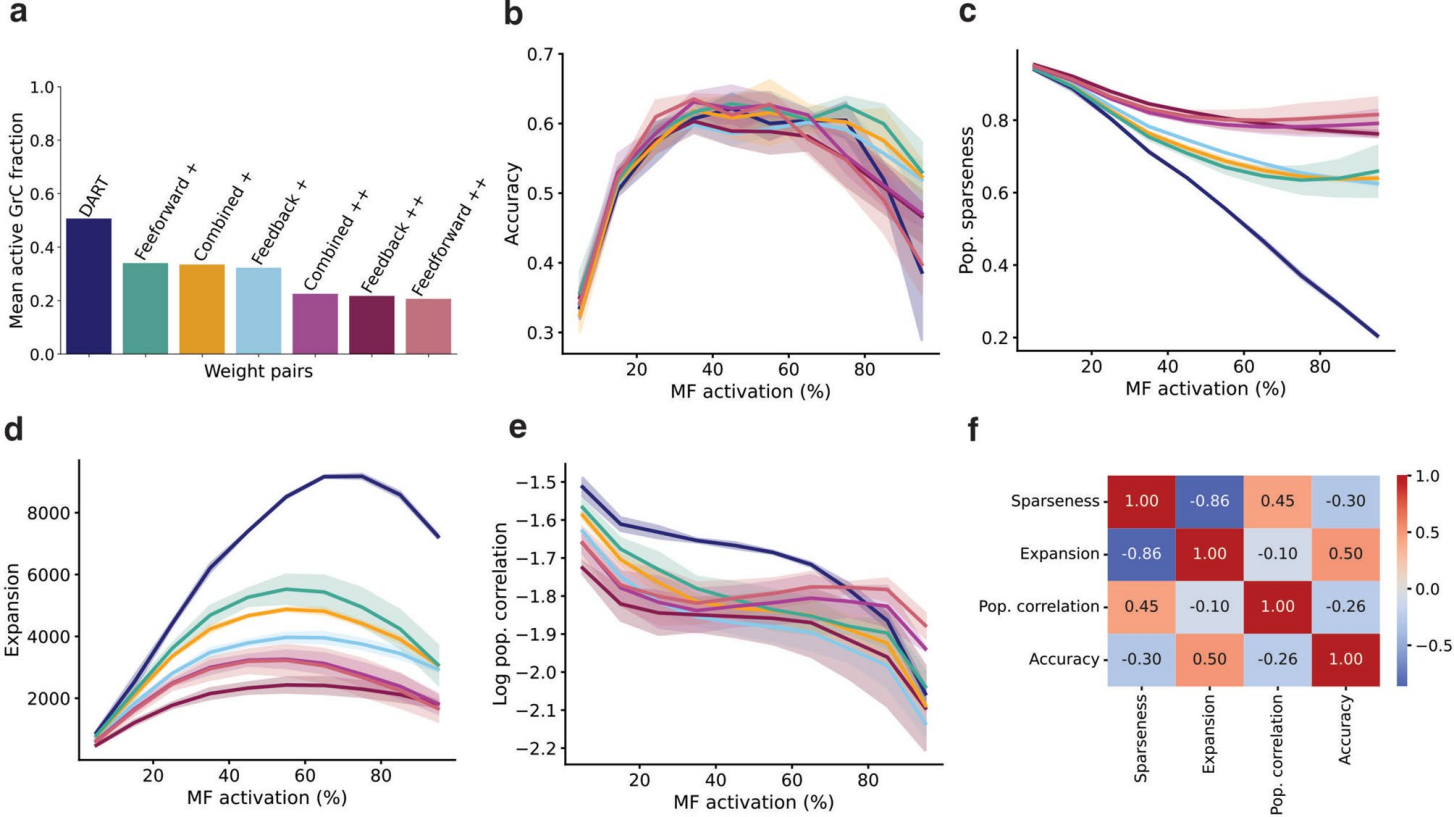
Moderate inhibition preserves pattern separation by balancing sparseness, correlation and coding expansion. (**a**) Average active GrC fraction across simulations for each configuration of weights MF—GoC and GrC—GoC. (**b**) Accuracy for the test set after 500-epoch-learning as a function of the input active fraction for mossy fibers for each pair of weights MF—GoC and GrC—GoC. Shading represents the confidence interval (95%). (**c**) Same as (**b**), for the population sparseness. (**d**) Same as (**b**), for the expansion of the coding space. (**e**) Same as (**b**), for the population correlation. (**f**) Correlation matrix among the different metrics and the classification accuracy.

Having established that inhibition influences pattern separation in the cerebellar granular layer, our final goal was to explore the mechanisms underlying the contribution of Golgi cell-mediated inhibition to this process. Since our model supports separate modulation of the feedback and the feedforward inhibitory loops (see Methods for additional details on synaptic weights), we tested several inhibition levels (Fig. 7a): one case with inhibition completely blocked (the previously mentioned DART case), three medium inhibition scenarios (targeting feedforward, feedback and both pathways), and three high inhibition scenarios (again distributed along feedforward, feedback and both pathways). As previously commented, inhibition had the greatest impact under high input activity conditions, with moderate levels of inhibition preserving performance accuracy most effectively (Fig. 7b).

We evaluated three potential effects that inhibition may have on GrC activity: population sparseness, expansion of the coding space (total variance) and population correlation (see Methods). We first analyzed population sparseness (Fig. 7c). In absence of inhibition (DART case), sparseness declined uniformly with increasing input activity. In contrast, inhibition showed effectiveness on maintaining sparseness levels even at high input levels. No significant differences can be extracted among the isolated feedforward, feedback and combined conditions.

We then evaluated the influence of inhibition on coding space expansion and its relationship with accuracy (Fig. 7d). The coding space exhibited greater expansion without inhibition (DART) than in the inhibited conditions. Notably, feedforward-based inhibition configurations resulted in more expansion than feedback-based inhibition. However, at high levels of input activity, coding expansion declined across all scenarios, likely due to saturation in the DART condition and excessive suppression (less fluctuations) in the highly inhibited cases.

Inhibition is also known to decorrelate neural activity. In line with this, most inhibitory conditions led to lower population correlations (or higher decorrelations) compared to the DART case, except under strong feedforward inhibition or combined loops at high input levels (Fig. 7e), where correlation increased due to excessive inhibition that silenced most granule cells. Among different configurations, feedback inhibition was more effective than feedforward in reducing correlations. Despite this, when correlating these metrics with accuracy (Fig. 7f), we found that neither sparseness nor decorrelation explained performance as well as coding expansion. Still, no single metric predicted accuracy reliably, but together they provide insight into how inhibition may shape pattern separation in the granule cell layer.

## Discussion

By developing a biologically detailed computational model of the cerebellar granular layer, we present a powerful tool for investigating the key principles underlying information representation within the granule cell population. Our model incorporates anatomically and physiologically grounded features^12,13^—such as neuron ratios, synaptic connectivity and axonal and dendritic length—which are critical for simulating realistic sensory stimulation. The model robustly reproduces granule cell responses to both auditory and somatosensory stimuli, capturing experimentally observed effects^28^ under varying inhibitory conditions.

Beyond faithfully reproducing experimental findings, we verify—by coupling the model output to a downstream decoder—that pattern separation performance in granule cells improves relative to mossy fiber inputs. Importantly, our model shows that synaptic inhibition from Golgi cells enhances the efficiency of the representations, particularly under high-complexity input conditions, reducing firing rates while preserving transmission of information to Purkinje cells.

Previous studies have identified expansion of coding space and correlations, rather than sparse activity, as primary contributors to pattern separation^20^. Moreover, inhibition has been shown to shape sensory input representations by sparsening and decorrelating granule cell activity^8–11^. In line with these findings, our results offer new insights by selectively manipulating the strength and type of inhibition, revealing how different inhibitory configurations modulate population sparsening, expansion and input decorrelation. Moderate inhibition—regardless of whether it targeted feedforward, feedback, or both pathways—was most effective in maintaining accurate performance. Although inhibition promoted sparseness and reduced correlations, it constrained the expansion of the coding space compared to the condition without inhibition, suggesting a trade-off between stability and representational diversity. Our results support prior work highlighting the role of feedback inhibition in promoting population decorrelations^10,11^. In contrast, we found that feedforward inhibition is particularly important for maintaining the coding space expanded, which appears to more directly enhance pattern separation performance. Overall, our findings suggest that inhibition supports pattern separation through a combination of mechanisms, rather than relying on a single dominant factor. Together, these results highlight the utility of the model, not only in replicating known phenomena, but also in uncovering how cerebellar microcircuit properties can contribute to information encoding and pattern recognition.

Our main goal was to create a general and adaptable framework. The model integrates the anatomical organization of the cerebellar granular layer, while maintaining a reduced scale to minimize computational cost. Input patterns are customizable and neural activity is implemented using leaky integrate-and-fire models^27^ to balance realism with efficiency. The resulting activity is evaluated using an explicit decoder, enabling direct assessment of classification performance and quality of information representation. In this way, our model fills a gap between detailed reconstructions and highly abstract networks, offering a robust platform for exploring diverse cerebellar functions.

A key strength of our model lies in its versatility and adaptability to a wide range of research questions. Most parameters can be modified to suit specific experimental or theoretical scenarios, including the volume size, the connectivity configurations, the nature of input patterns, and the balance of excitation and inhibition across mossy fibers, granule cells and Golgi cells. This tunability allows the model to serve as a flexible platform for investigating circuit behavior, coding strategies, and learning features in the cerebellum and beyond. Importantly, replicating experimental data is not just a means to validate our model, but also a powerful way to prove the underlying mechanisms that could generate those observations—such as strength of inhibition, the proportion of active mossy fibers, or the spatial distribution of the input patterns—offering insights that are often inaccessible through experiments alone. To promote reproducibility and collaborative development, the entire tool is openly available to the research community. This accessibility facilitates the extension and adaptation of the model by other groups, fostering broader applications across computational neuroscience.

While our model provides a biologically grounded and flexible framework for investigating cerebellar granular layer dynamics, several limitations remain that point to promising directions for future development. First, the current implementation does not yet incorporate gap junctions between Golgi cells, which have been shown to play a key role in synchronizing inhibitory activity and shaping the spatial and temporal organization of granule cells responses^31–34^. Additionally, some neuronal populations of the granular layer—such as Lugaro cells and unipolar brush cells—are not yet included, potentially limiting the model capacity to capture the full diversity of interactions within the microcircuit. Tonic inhibition, another important modulatory factor in granule cell excitability, is also absent from the current version and is yet to be implemented. Due to computational constraints, we conducted our tests on relatively small-scale compared to other large-scale simulations of the cerebellar network^13,17–19^. Although the model is scalable, the systematic simulation of high-dimensional input patterns imposes a significant computational load and, for running large-scale versions with dense activity patterns as we did, high performance computing clusters are typically required.

To illustrate the potential of the framework, we outline several directions for its application in studying cerebellar dynamics. Recent experimental evidence shows that granule cell activity in the cerebellar cortex is highly contextual, multimodal, and anatomically clustered^28,35^, forming a dense and redundant coding scheme that challenges the sparse representations proposed by Marr and Albus^36–38^. This dense activity is regulated by Golgi cells, which act as threshold for mossy fiber input and granule cell responses^22,37,39–41^ and seem to be pivotal in shaping their spatial organization^13,42–45^. Our model enables the simulation and study of this scenario via spatially correlated input patterns of high activity and tunable levels of synaptic inhibition. Furthermore, the model allows for the isolation and independent analysis of feedforward and feedback inhibitory pathways, which arise from Golgi cell dendritic architecture and have been linked to temporal precision and oscillatory patterns of granule cell activity^17,39,41,46,47^.

The model could also support future studies on how structured connectivity and plasticity influence information processing in the cerebellar cortex. Interestingly, despite dense widespread granule cell activity, synaptic connectivity between mossy fibers and granule cells remains sparse^29,48,49^. Moreover, it has been shown that cerebellar circuits exhibit non-random, structured connectivity—a feature shown to enhance network resilience to noise^48^. This property can be systematically explored within our framework by customizing synaptic connectivity prior to simulation. Finally, the model serves as a platform to study how different forms of synaptic plasticity—including GABAergic modulation of LTP and LTD^50–52^, as well as STDP processes influenced by theta-band oscillations^53–56^—shape pattern separation and sensory input transformation in the granular layer. We hope this model will serve as a starting point for future studies aimed at testing new hypotheses about sensory encoding, learning, and circuit-level computation in the cerebellar cortex.

## Methods

### Anatomical model

The three-dimensional model of the cerebellar granular layer was built following other biologically realistic models procedure^12,13^, with two main basic steps: first, the generation of the neuron positions and, second, the generation of the connections between them. Three populations were considered in this work: the glomeruli, the granule cells (GrC) and the Golgi cells (GoC). Please, note that glomeruli and mossy fibers (MF) are considered equivalent in all this manuscript as each MF targets a single glomerulus and vice versa.

For each neural population, parameters such as density, radius of the dendrites/axons and size were derived from literature^12,13,57^. Cells were positioned through an *ad-hoc* algorithm (Bounded Self-Avoiding Random Walk) according to reference^13^, inside a cube of side size 100 um. This algorithm ensures that all the cells are uniformly distributed along the whole cube while maintaining certain randomness on the exact positioning. The number of elements generated for our model varied slightly depending on the initial seed, around 300 MF, 3700 GrC and 8 GoC.

Connectivity in the model was established according to reference^13^. The connections considered in this model were: from MF to GrC (excitatory connection), from MF to GoC (excitatory connection), from GrC to GoC (excitatory connection), through ascendent axons (AA) and through parallel fibers (PF), and from GoC to GrC (inhibitory connection). In order to avoid possible boundary effects, when connecting neurons, ends of all cube axes were considered as a continuity of their beginnings. Parameters relevant for the connection were divergence and convergence ratios and geometric constraints such as dendrites and axon lengths and their spreading directions. A more detailed explanation of each implemented connection can be found in the Supplementary Material.

### Spatially correlated input patterns

MF activity patterns were created, following steps from^20^, using a method based on dichotomized gaussian models^26^ that generates binary vectors given two parameters: the specified average values were the average MF active fraction and the correlation coefficients represented a Gaussian distribution of distances between MFs with the standard deviation (σ) parametrized by a chosen correlation radius. Therefore, for a larger sigma, correlation reached further MF in the cube, forming patterns with clusters of active MF. On the contrary, smaller sigma values resulted in active MF in patterns more uniformly distributed in the cube, as shown in Fig. 1d (see also Supplementary Fig. S2). In the results section, the used sigma value is indicated for each specific experiment.

### Neuron models and simulations

Once cell positions, connections and input patterns were established, simulations were run using the EDLUT simulator^27,58^. MFs were modeled as Poisson processes with firing rate determined by the generated binary activity patterns described above (50 Hz if the MF was activated, silent -0 Hz- otherwise). In order to build a biologically plausible as well as computationally efficient model, GrC and GoC were modeled using Leaky Integrate-and-Fire (LIF) models. In the LIF model, the neural state is characterized by the membrane potential (*V*_*m-c*_) defined by the differential equation (Eq. A1). This equation includes the effect of chemical synapses [α-amino-3-hydroxy-5-methyl-4-isoxazolepropionic acid (AMPA) and gamma-aminobutyric acid (GABA) receptors] and the resting conductance (*G*_*rest*_),

A1 $$C_{m} \cdot \frac{{dV_{m - c} }}{dt} = g_{AMPA} (t) \cdot \left( {E_{AMPA} - V_{m - c} } \right) + g_{GABA} (t) \cdot \left( {E_{GABA} - V_{m - c} } \right) + g_{rest} \cdot \left( {E_{rest} - V_{m - c} } \right)$$

where *C*_*m*_ denotes the membrane capacitance, *E*_*AMPA*_ and *E*_*GABA*_ stand for the reversal potential of each synaptic conductance, and *E*_*rest*_ represents the resting potential (with *G*_*rest*_ being the conductance responsible for the passive decay term toward the resting potential). Conductances *g*_*AMPA*_ and *g*_*GABA*_ integrate all the contributions received by each receptor type (AMPA and GABA) through individual synapses and are defined as decaying exponential functions which provide reasonable accuracy at a low computational cost (Eq. A2),

A2 $$ \begin{array}{*{20}c} {g_{AMPA} (t) = \left\{ {\begin{array}{*{20}c} {0,} \\ {g_{GAPA} (t_{0} ) \cdot e^{{{{ - (t - t_{0} )} \mathord{\left/ {\vphantom {{ - (t - t_{0} )} {\tau_{AMPA} }}} \right. \kern-0pt} {\tau_{AMPA} }}}} ,} \\ \end{array} \begin{array}{*{20}c} {t \le t_{0} } \\ {t > t_{0} } \\ \end{array} } \right.} \\ {g_{GABA} (t) = \left\{ {\begin{array}{*{20}c} {0,} \\ {g_{GABA} (t_{0} ) \cdot e^{{{{ - (t - t_{0} )} \mathord{\left/ {\vphantom {{ - (t - t_{0} )} {\tau_{GABA} }}} \right. \kern-0pt} {\tau_{GABA} }}}} ,} \\ \end{array} \begin{array}{*{20}c} {t \le t_{0} } \\ {t > t_{0} } \\ \end{array} } \right.} \\ \end{array} $$

where* t* represents the simulation time whilst *t*_*0*_ denotes the arrival instant of an input spike, *g*_*AMPA*_ stands for the AMPA receptor which provides excitation, and *g*_*GABA*_ stands for the GABA receptor-mediated conductance, which provides inhibition. Finally, *τ*_*AMPA*_ and *τ*_*GABA*_ are the decaying time constants of each receptor type. Synapses were simplified using constant synaptic weights. The model parameters^59^ for each population can be found in Supplementary Materials (see Supplementary Table S1). Note that the main aim of this work was to study the impact of the network topology in the information encoding. In this way, we have preferred to maintain the neuron models as simple as possible and we have not modeled the effect of other mechanisms, such as NMDA, spillover conductances, short-term plasticity or intrinsic adaptation.

The network synaptic weights have been derived by establishing the weight of MF—GrC and then adjusting the others to calibrate network responses (see Supplementary Table S2). The peak AMPA conductance for MF—GrC was set to 4.0 nS, distributed among the four synapses of each GrC (4.0/4 = 1.0 nS), ensuring that, on average, and in absence of inhibition, at least three MFs needed to fire in close temporal proximity to generate spikes in that GrC^20^. Peak GABA conductance for GoC—GrC synapse was fixed to 0.5 nS aiming to produce visible impact on GrC firing rate, and the inhibition level was indirectly increased or decreased by excitation weights from MF—GoC (feedforward) and GrC—GoC (feedback) synapses. The ratio between these two excitatory weights was chosen by running preliminary simulations varying the weights and checking which combination of weights resulted in similar GrC firing rates (see Supplementary Fig. S4) and reproduced similar response probabilities to those reported in^28^. Unless otherwise mentioned in text, MF—GoC weight was set to 0.1 nS, while GrC—GoC was set to 0.015 nS. In Fig. 6, “No Inh” refers to w (MF—GoC) = 0.0 and w (GrC—GoC) = 0.0, replicating DART effect; “Inh + ” refers to w (MF—GoC) = 0.1 and w (GrC—GoC) = 0.005; and finally, “Inh +  + ” refers to w (MF—GoC) = 0.1 and w (GrC—GoC) = 0.015.

### Stimulation implementation

For both sensory-evoked auditory and somatosensory stimuli, we found that medium fractions of active mossy fibers—ranging from 35 to 65%—produced similar outcomes. For each simulation, we selected the fraction within this range that best matched the experimental results from^28^. Firing rates were also tuned to align with experimental response probabilities reported in^28^, where granule cell activation probabilities were approximately 0.15 for auditory stimuli and 0.06 for somatosensory stimuli under control conditions. In our model, a mossy fiber firing rate of 50 Hz yielded a granule cell response probability of 0.13, which we selected for auditory stimuli. Similarly, a 100 Hz input resulted in response probability of 0.08 and was chosen for somatosensory stimuli. For experiments in Fig. 2, we additionally differentiated stimulus types by the spatial correlation of input patterns. Auditory stimuli were modeled with more spatially dispersed activity (σ = 5 µm), while somatosensory stimuli were more clustered (σ = 40 µm), reflecting observations from the first experiment in^28^.

### Categorization of granule cells

In results from Fig. 4, categories for *facilitated*, *unchanged*, *suppressed* and *emergent* for each GrC were chosen as follows: firstly, the GrC mean response in both individual patterns was calculated and the higher one was assigned as a threshold. The higher activity of the GrC in both individual patterns was also saved as a maximum. Then, the categories were established: if, for both individual patterns and the combinated pattern, the activity of the GrC was below the threshold, it was categorized as *unchanged*; if, for both individual patterns, the activity of the GrC was below the threshold and for the combinated one was above, it was categorized as *emergent*; if, for one of the individual patterns, the activity of the GrC was above the threshold and for the combinated one was below, it was categorized as *suppressed*; if, for both individual patterns and the combinated pattern, the activity of the GrC was above the threshold but the combinated one was above the established maximum, it was categorized as *facilitated* and otherwise as *unchanged*.

### Data collection and classifier implementation

Following the methodology previously proposed in^20^, individual patterns were chosen as simulation inputs, while for studying the performance classification, sets of 640 patterns for each MF fraction (from 5 to 95% of activation) were used. Activity resulting from the simulations were collected as spike counts of each neuron for each pattern. A single layer perceptron decoder was trained to classify the 640 input patterns into 10 random classes. Random classification was chosen to ensure maximal overlap between patterns. The number of classes was chosen after previous experiments to produce difficult enough learning tasks, allowing comparison of different networks.

In our model, a Single-Layer Perceptron (SLP) was built with a softmax activation function, since the task involved multiclass classification. Softmax transforms the raw output scores into a probability distribution over the output classes. The chosen optimizer was stochastic gradient descent and since we were dealing with a classification problem, the loss was calculated via *categorical cross-entropy.* We tested learning rates of 0.1, 0.01, and 0.001 and found that 0.001 gave the most stable and best generalizing results. Likewise, we chose a batch size of 32 in order to avoid overfitting. We trained for up to 2000 epochs with early stopping (patience of 5), observing that validation accuracy exhibited a plateau around 500 epochs for the majority of MF active fractions and therefore establishing a training number of 500 epochs. The inputs consisted of either the raw number of MF or GrC spikes during the time that each pattern was presented (80 ms), discarding the initial stabilization period (10 ms). The chosen metric to evaluate the performance was *accuracy* (i.e. *correct classifications divided by total number of classifications*).

### Noise implementation

Noisy patterns were implemented to provide a more challenging classification for the SLP. Given a noise percentage, such as 20%, then 20% of random MFs were chosen for each pattern of a set of 640 patterns, and their activity was reset, with a probability of being active given by the average MF active fraction of the patterns set. This ensured the activity average of the set of patterns would remain constant. The perceptron inputs then consisted of the resulting noisy MF or GrC activity patterns. During the EDLUT simulation, each noisy pattern was presented 10 times (10 different random occurrences) for each training epoch. The obtained group of 6400 samples (640 patterns × 10 presentations) was divided into a training set (70% of samples), validation set (10% of samples) and test set (20% of samples). Unless otherwise indicated, the noise percentage used during simulations was 20%.

### Classification results analysis

To ensure the statistical significance of the classification results, the training was performed with 6 different initial seeds. The seeds influenced both the pattern generation and the network topology (including neuron positioning and connections). Average and standard deviations were calculated for the accuracy results. From this data, confidence intervals (CI) with a confidence level of 95% were obtained and represented in the plots. To compare mossy fibers and granule cells results (with and without inhibition), we computed paired sample *t*-tests to determine whether the mean difference between our two sets of observations was zero, with the null hypothesis therefore assuming that the true mean difference between the paired samples is zero.

### Mechanistic analysis

The mechanistic analysis was performed by studying granule cell activity with three different metrics: population sparseness, expansion of the coding space and population correlation. Population sparseness was measured as^20^:

$$Pop.\,sparseness = \frac{{N - \frac{{\left( {\mathop \sum \nolimits_{i} x_{i} } \right)^{2} }}{{\mathop \sum \nolimits_{i} x_{i}^{2} }}}}{N - 1}$$

where N is the number of neurons and ${x}_{i}$ is the ith neuron’s spike count. It was averaged over all activity patterns.

To quantify expansion of coding space, we used the total variance as in^20^, i.e. the sum of all variances:

$$Total\,variance = \mathop \sum \limits_{i} {\text{var}} \left( {x_{i} } \right)$$

We defined the population correlation as^20^:

$$Pop. correlation = \frac{N}{N - 1}\left( {\frac{{\max \left( {\sqrt {\lambda_{i} } } \right)}}{{\mathop \sum \nolimits_{i} \sqrt {\lambda_{i} } }} - \frac{1}{N}} \right)$$

where ${\lambda }_{i}$ are the eigenvalues of the covariance matrix of the activity patterns. To ensure the statistical significance, the different metrics were calculated for 3 different initial seeds. In Fig. 7, labels used for each pair of synaptic weights are reported in Table 1.

**Table 1:** Synaptic weights used for each pair of weights MF—GoC and GrC—GoC.

Label	W(MF-GoC) (nS)	W(GrC-GoC) (nS)
DART	0.0	0.0
Feedforward +	0.2	0.0
Feedback +	0.0	0.03
Combined +	0.1	0.015
Feedforward + +	0.3	0.0
Feedback + +	0.0	0.06
Combined + +	0.2	0.015

## Supplementary Information

Supplementary Information.

## Data Availability

Models and scripts for running and analyzing simulations are available at https://github.com/EduardoRosLab/GCL-activity-analysis.
