# Supplementary material for: A computational model of the cerebellar granular layer calibrated to experimental data for studying inhibition and sensory encoding
Source: Sci Rep. 2025 Nov 25;15:41788. doi: 10.1038/s41598-025-25727-5 (PMC12647829; doi:10.1038/s41598-025-25727-5)
Supplement: Supplementary file 1 — Supplementary Information. [file 41598_2025_25727_MOESM1_ESM.docx]

**Supplementary Information**

*A computational model of the cerebellar granular layer calibrated to experimental data for studying inhibition and sensory encoding*

María P. Tirado^1,*^, Eva M. Ortigosa^1^, Eduardo Ros^1^, Jesús A. Garrido^1^

^1^Department of Computer Engineering, Automation and Robotics. University of Granada, Spain

^*^Corresponding author

E-mail: [mariatirado@ugr.es](mailto:mariatirado@ugr.es)

**Supplementary Methods**

**Anatomical model connections.**

Each synaptic connection has been shaped following already known experimental values [1, 2]. Information about each connection is detailed below:

**MF - GrC excitatory connection:**

Connections were generated if the distance between the GrC and MF was less than the GrC dendrites maximum length, 40 um. Since each granule cell receives on average ~4 synaptic inputs (each dendrites on average), if the number of mossy fibers that satisfy the condition was more than the number of dendrites, the four closer ones were chosen. The resulting mean distance between GrC and MF, mean granule dendritic length, was around 12 um (the experimental mean dendritic length is 13.6 µm) [1, 2]. In our model, each MF reaches on average 50 GrC dendrites, while the experimental value is ~53 (see **Supplementary** **Fig. S1a**).

**MF - GoC excitatory connection:**

GoC basolateral dendrites spread forming a semi sphere of 50 µm radius under the GoC soma. We connected each MF located into this volume exclusively to that GoC  [1]. In our model, each MF reaches on average ~2.2 GoC (see **Supplementary Fig. S1b**).

**GOC - GRC inhibitory connection:**

The axon of GoCs expand up to 150 µm on both x- and y-axes (sagittal plane), in a spiral way, while remaining narrow on the z-axis. Each axon can form synapses with a maximum of 40 separate MFs, thus in the absence of enough postsynaptic cells inside this volume, we connected the nearest 40 ones. GoCs will inhibit all GrCs connected to those MFs [1]. In our model, each GoC reaches on average ~1380 GrCs (See **Supplementary Fig. S1c**)

**GRC - GOC excitatory connection:**

AA: GoCs basolateral and apical dendrites form a sphere around the soma, with an arborization of 50 µm radius, with the latter going up to reach the molecular layer.

Ascending axons (AA) of GrCs will form connections if they cross that volume. In addition, even GrC cells are considered whose soma is situated into a virtual cylindrical volume where the base is a circle centered on the GoC soma and with radius of 50 µm, while the height is equal to that of the corresponding potential postsynaptic GoC y-coordinate. Each ascending axon can establish only one connection with a GoC, while a single Golgi cell can have no more than 400 connections, selected with a probability-based rule [1].

PF: GoC apical dendrites connect also with the parallel fibers originated from the same GrC that form connections with their ascending axons, together with other PF randomly chosen that cross the area formed by the apical dendrites, with the goal of reaching a total convergence value of 1600 [1].

In our model, each GrC reaches on average ~3 GoCs (See **Supplementary Fig. S1d**).

**Neuron models and simulations.**

Granule cells and Golgi cells were modeled using Leaky Integrate-and-Fire (LIF) models implemented in the EDLUT simulator [3, 4]. The model parameters are presented in **Supplementary Table S1**.  The synaptic weights for each connection are presented in **Supplementary Table S2**.

| **Parameter** | **GrC** | **GoC** |
| --- | --- | --- |
| **Membrane capacitance (C_m_; pF)** | 2 | 50 |
| **Firing threshold (V_m_; mV)** | -40 | -50 |
| **Resting potential (E_rest_; mV)** | -65 | -65 |
| **Excitatory reversal potential (E_AMPA_; mV)** | 0 | 0 |
| **Inhibitory reversal potential (E_GABA_; mV)** | -65 | -65 |
| **Resting conductance (G_rest_; nS)** | 0.2 | 3 |
| **Resting time constant (τ_rest_; ms)** | 10 | 16.7 |
| **AMPA receptor time constant (τ_AMPA_; ms)** | 0.5 | 0.5 |
| **GABA receptor time constant (τ_GABA_; ms)** | 10 | 10 |
| **Refractory time (τ_ref_; ms)** | 1.5 | 1.5 |

**Supplementary Table S1**. Parameters used for each neuron population extracted from [5].

| **Connection** | **Synaptic weight (nS)** |
| --- | --- |
| **MF - GrC** | 1.0 |
| **GrC - GoC** | 0.015 |
| **MF - GoC** | 0.1 |
| **GoC - GrC** | 0.5 |

**Supplementary Table S2**. Synaptic weights for each connection, based on preliminary simulations.

**Supplementary References**

[1] Casali, S., Marenzi, E., Medini, C., Casellato, C., & D'Angelo, E. Reconstruction and simulation of a scaffold model of the cerebellar network. *Front. Neuroinform.*, **13**, 444802 (2019).

[2] Solinas, S., Nieus, T., & D’Angelo, E. A realistic large-scale model of the cerebellum granular layer predicts circuit spatio-temporal filtering properties. *Front. Cell Neurosci*. **4**, 903 (2010).

[3] Ros, E., Carrillo, R., Ortigosa, E. M., Barbour, B., & Agís, R. Event-driven simulation scheme for spiking neural networks using lookup tables to characterize neuronal dynamics. *Neural Comput.*, **18(12)**, 2959-2993 (2006).

[4] Luque, N. R., Garrido, J. A., Naveros, F., Carrillo, R. R., D'Angelo, E., & Ros, E. Distributed cerebellar motor learning: a spike-timing-dependent plasticity model. *Front. Comput. Neurosci.*, **10**, 17 (2016).

[5] Garrido, J. A., Ros, E., & D’Angelo, E. Spike timing regulation on the millisecond scale by distributed synaptic plasticity at the cerebellum input stage: a simulation study. *Front. Comput. Neurosci.*, **7**, 64 (2013).


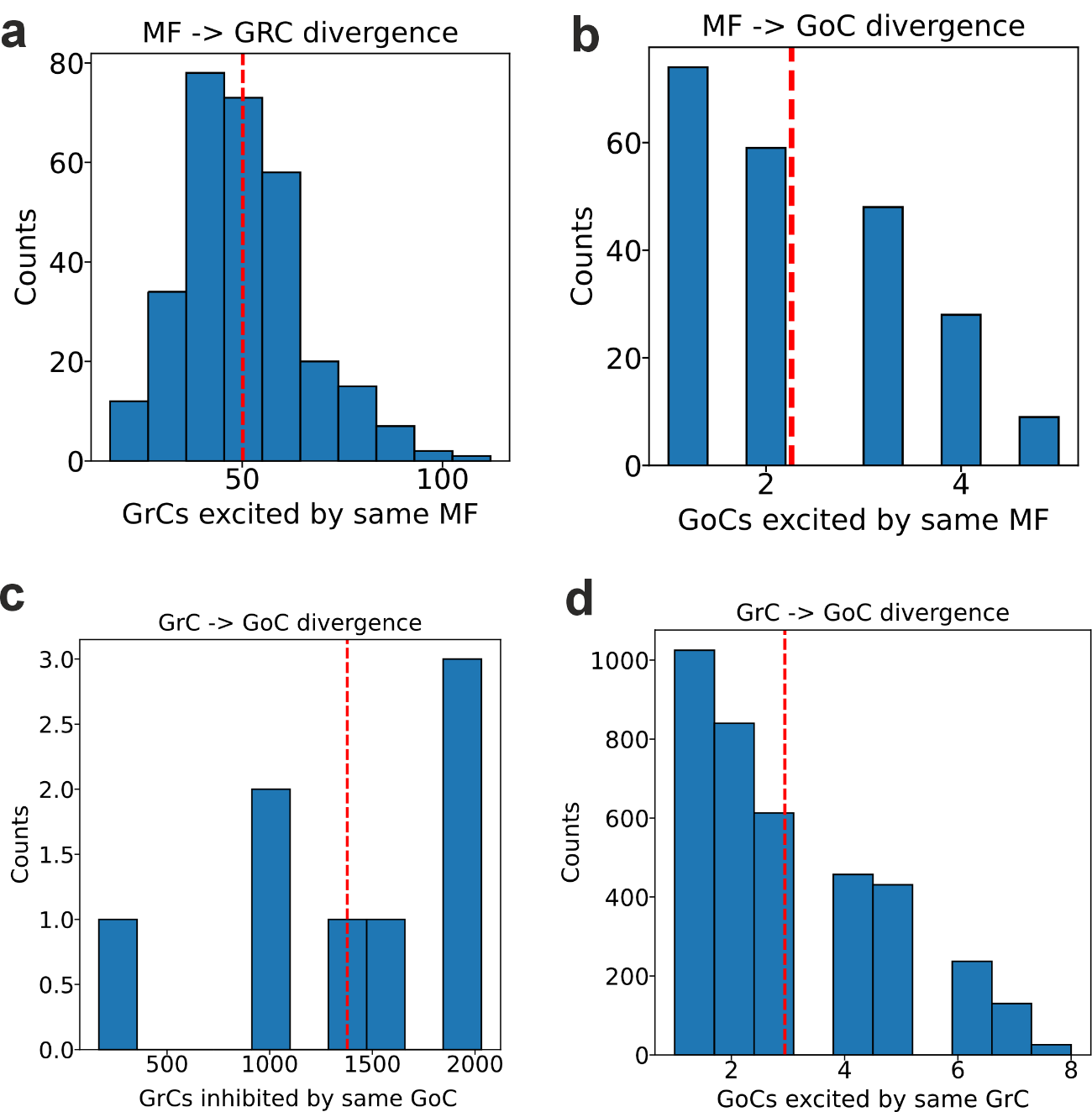


**Supplementary Fig. S1 Anatomical model statistics. a.** Histogram of MF convergence to GrCs. **b.** Same as **a**, for MF convergence to GoCs. **c.** Same as **a**, for GrCs divergence to GoC in the inhibitory synapse. **d.** Same as **a**, for GrC divergence to GoCs in the excitatory synapse.


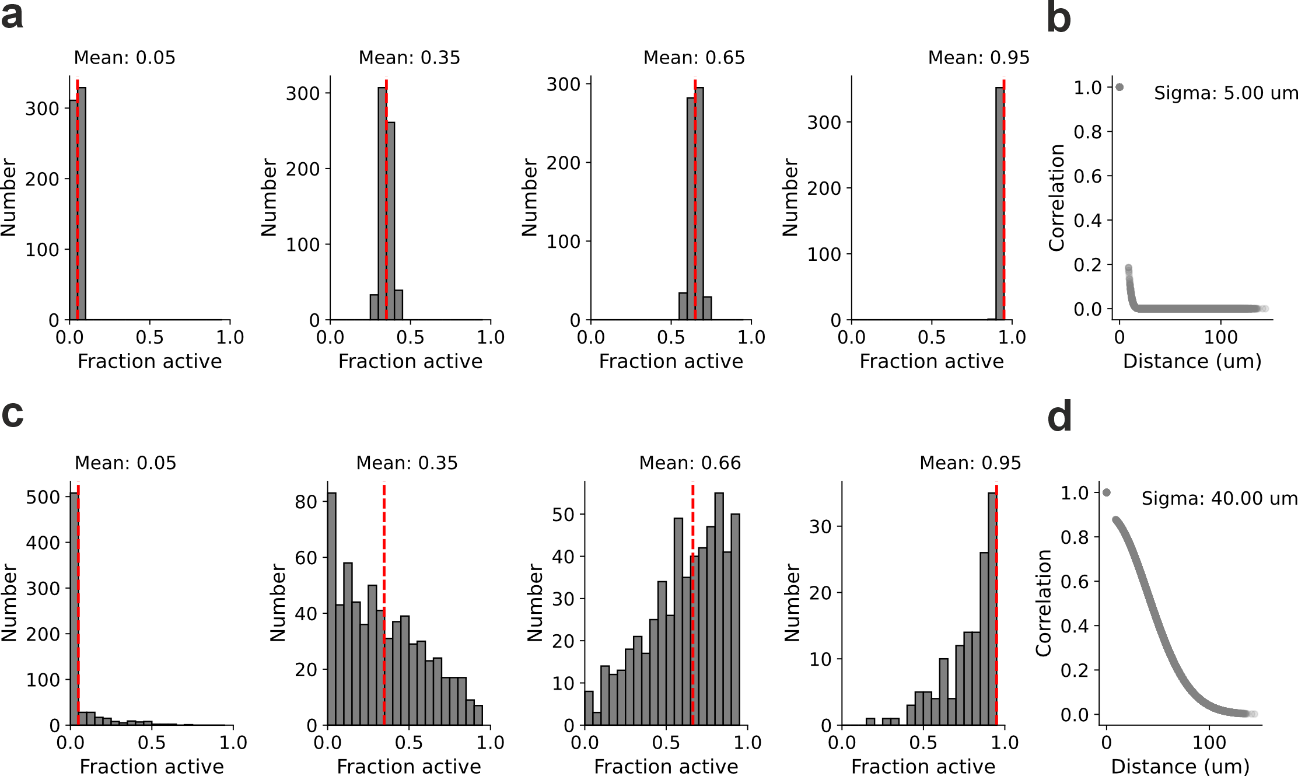


**Supplementary Fig. S2 MF input statistics. a.** Histograms of the fraction of active MFs over different activity patterns with a correlation radius of σ = 5 um. Each histogram corresponds to a different average fraction of active MFs (from left to right: 0.05, 0.35, 0.65 and 0.95). **b.** Correlation between MF pairs plotted against distance between them, for a correlation radius of σ = 5 um. **c.** Same as **a**, with a correlation radius of σ = 40 um. **d.** Same as **b** for a correlation radius of  σ = 40 um.


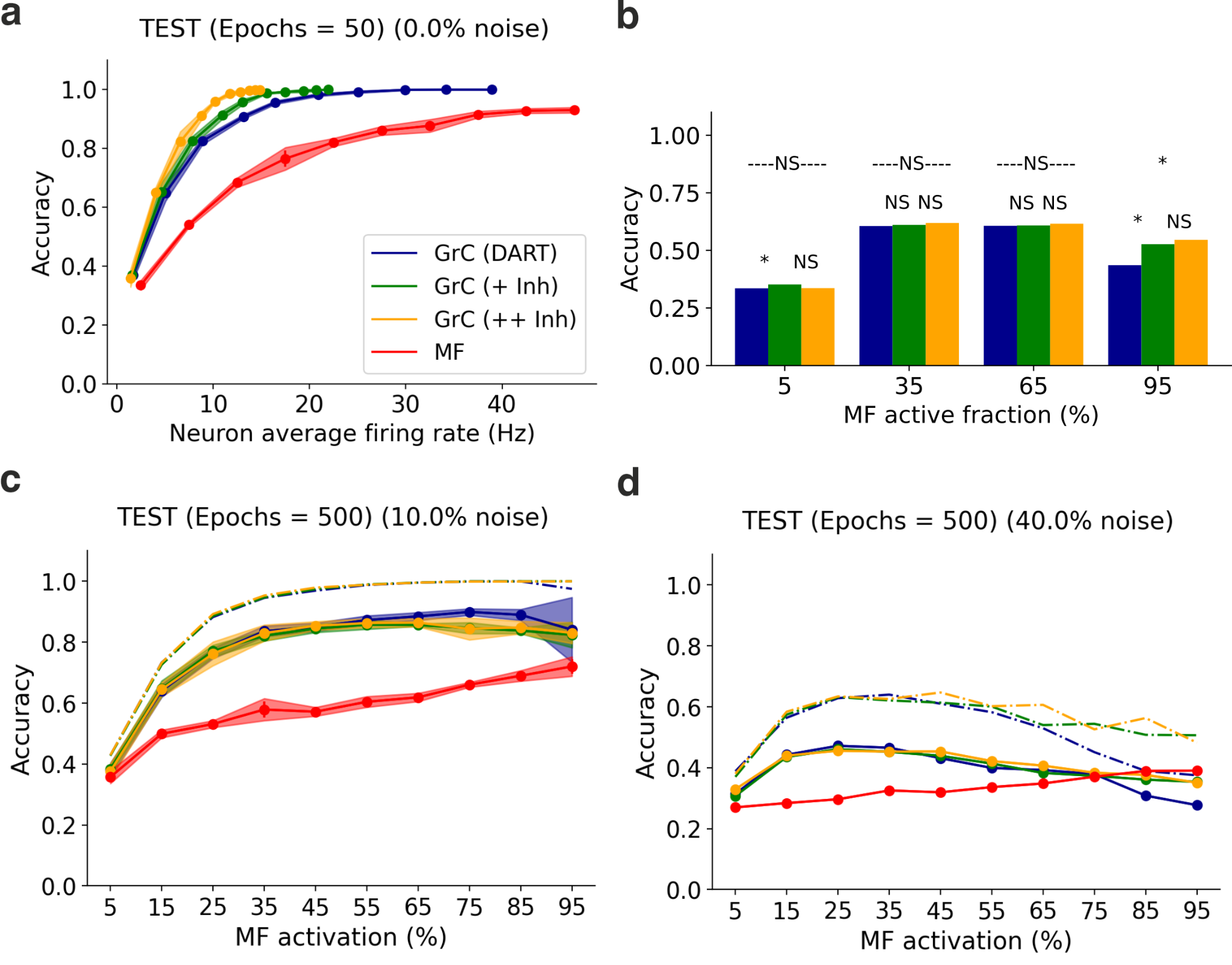

**Supplementary Fig. S3 Effect of inhibition in accurate recognition of noisy patterns. a.** Accuracy test results after 50-epoch-learning for a case with 0% noise, as a function of the GrC and MF average firing rate and different levels of inhibition. Shading represents the confidence interval (95%). **b.** Histograms of accuracy test results for 4 different active fractions (0.05, 0.35, 0.65, 0.95), for the case of 500 epochs and 20% noise, showing the p-values obtained between the GrC configurations.  **P* < 0.05, paired *t* test. *NS*, not significant. **c.** Accuracy for the test set with 10% of noise after 500-epoch-learning as a function of the input active fraction for mossy fibers and different levels of inhibition. Shading represents the confidence interval (95%). Dash-dotted lines represent the accuracy obtained with the training set with different levels of inhibition. **d.** Accuracy for the test set with 40% of noise after 500-epoch-learning as a function of the input active fraction for mossy fibers and different levels of inhibition. Dash-dotted lines represent the accuracy obtained with the training set with different levels of inhibition. 40 presentations of each pattern were used and only one seed, due to long computational time.


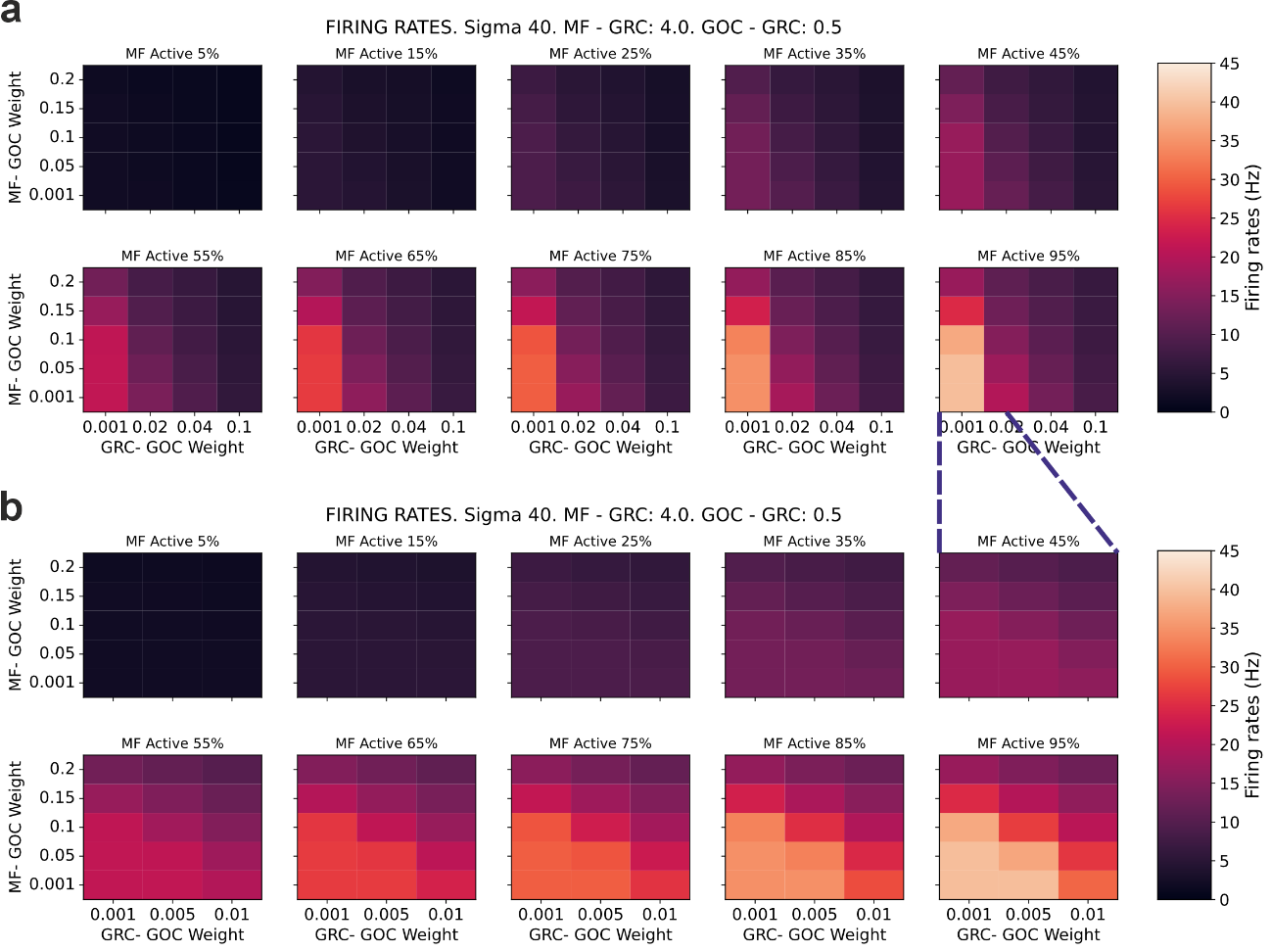


**Supplementary Fig. S4 Study of GrC population firing rates generated by different values of MF - GoC and GrC - GoC weights. a.** Heatmaps of GrCs firing rates in Hz for each MF active fraction from 0.05 to 0.95 for each combination of weight parameters. Notice how the weight combination of w(MF - GoC) = 0.2 and w(GrC - GoC) = 0.001 generates the same firing rates as w(MF - GoC) = 0.001 and w(GrC - GoC) = 0.02). **b.** Same as **a**, narrowing the range of GrC - GoC weights.


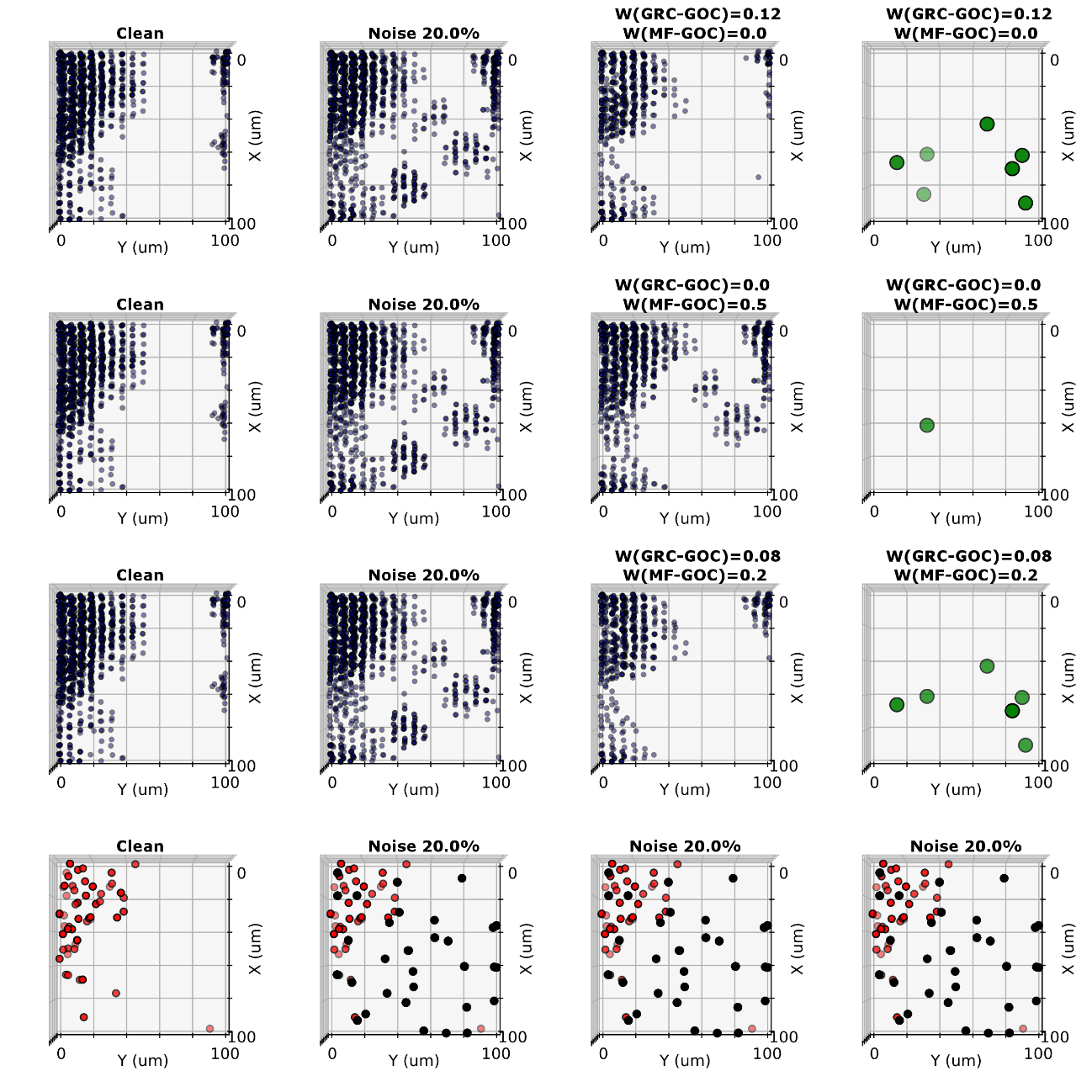


**Supplementary Fig. S5 Visualizations of an example of the GoC inhibitory action in GrC pattern representations.** Each square represents the cube from the apical axis perspective (Z). First row, from left to right: GrCs activity generated by a raw MF input pattern; GrCs activity generated by same input pattern, altered with 20% of noise; GrCs activity under the influence of inhibition from the feedforward loop (GrC - GoC synapse); GoCs activity generated by GrC - GoC synapses. Second row, same as first row with GrCs activity under the influence of inhibition from the feedback loop (MF - GoC synapse). Third row, same as first row with GrCs activity under the influence of both inhibitory loops, feedback and feedforward. Fourth row, from left to right: MF activity pattern; MF activity pattern altered with 20% noise, with noisy MF inputs shown as black dots.


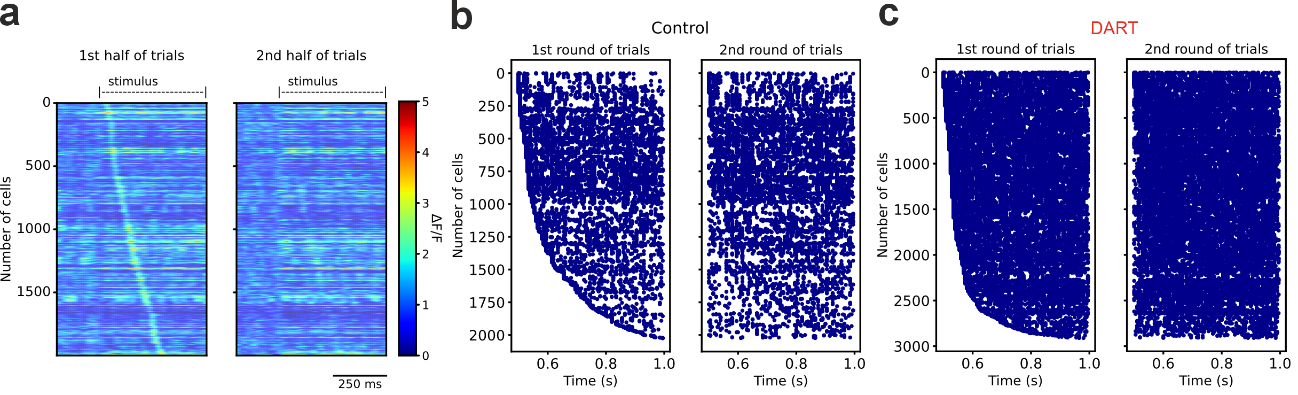


**Supplementary Fig. S6 Study of the temporal consistency in GrCs pattern representations a.** Left, mean stimulus activity on the first half of trials for all cells ordered by peak response time (n = 2000). Right, same as on left for the second half of trials, ordered according to peak responses during the first half. **b**. Left, spikes responses on the first half of trials for all cells ordered by first spike time (n = 3159). Right, same as on left for the second half of trials, ordered according to first spike times during the first half. Control case with inhibition type “Inh ++” (See Methods) and 20% noise. **c**. Same as **b**, for the DART case with 20% noise (n = 3760).


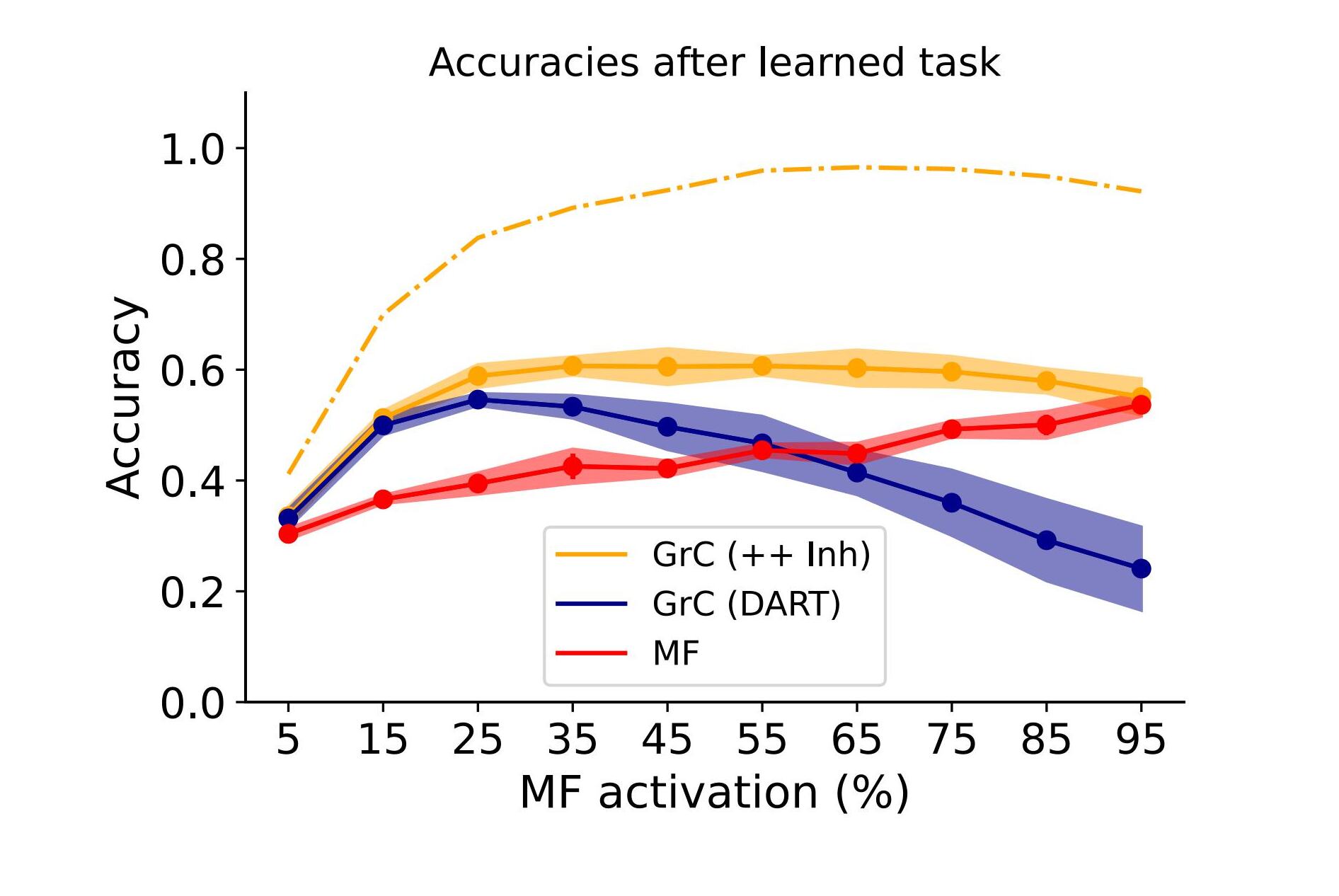


**Supplementary Fig. S7 Inhibition is necessary to express a learned association.** Accuracy for the test set with 20% of noise after 500-epoch-learning as a function of the input active fraction for mossy fibers. Shading represents the confidence interval (95%). Dash-dotted lines represent the accuracy obtained with the training set.
